# Heterogeneous gene expression during early arteriovenous fistula remodeling suggests that downregulation of metabolism predicts adaptive venous remodeling

**DOI:** 10.1038/s41598-024-64075-8

**Published:** 2024-06-10

**Authors:** Yuichi Ohashi, Clinton D. Protack, Yukihiko Aoyagi, Luis Gonzalez, Carly Thaxton, Weichang Zhang, Masaki Kano, Hualong Bai, Bogdan Yatsula, Rafael Alves, Katsuyuki Hoshina, Eric B. Schneider, Xiaochun Long, Rachel J. Perry, Alan Dardik

**Affiliations:** 1 Vascular Biology and Therapeutics Program, Yale School of Medicine, New Haven, CT USA; 2 Department of Surgery, Yale School of Medicine, New Haven, CT USA; 3 Division of Vascular Surgery, Department of Surgery, The University of Tokyo, Tokyo, Japan; 4 Department of Surgery and Science, Graduate School of Medical Sciences, Kyushu University, Fukuoka, Japan; 5 Department of Cardiovascular Surgery, Tokyo Medical University, Tokyo, Japan; 6 Department of Surgery, Center for Health Services and Outcomes Research, Yale School of Medicine, New Haven, CT USA; 7 Vascular Biology Center, Medical College of Georgia at Augusta University, Augusta, GA USA; 8 Department of Internal Medicine, Yale School of Medicine, New Haven, CT USA; 9 Department of Cellular and Molecular Physiology, Yale School of Medicine, New Haven, CT USA; 10 Surgical Service, Veterans Affairs Connecticut Healthcare System, West Haven, CT USA; 11 Yale School of Medicine, 10 Amistad Street, Room 437, PO Box 208089, New Haven, CT 06520-8089 USA

**Keywords:** Cardiovascular biology, Cardiovascular biology, Bioinformatics, Haemodialysis

## Abstract

Clinical outcomes of arteriovenous fistulae (AVF) for hemodialysis remain inadequate since biological mechanisms of AVF maturation and failure are still poorly understood. Aortocaval fistula creation (AVF group) or a sham operation (sham group) was performed in C57BL/6 mice. Venous limbs were collected on postoperative day 7 and total RNA was extracted for high throughput RNA sequencing and bioinformatic analysis. Genes in metabolic pathways were significantly downregulated in the AVF, whereas significant sex differences were not detected. Since gene expression patterns among the AVF group were heterogenous, the AVF group was divided into a ‘normal’ AVF (nAVF) group and an ‘outliers’ (OUT) group. The gene expression patterns of the nAVF and OUT groups were consistent with previously published data showing venous adaptive remodeling, whereas enrichment analyses showed significant upregulation of metabolism, inflammation and coagulation in the OUT group compared to the nAVF group, suggesting the heterogeneity during venous remodeling reflects early gene expression changes that may correlate with AVF maturation or failure. Early detection of these processes may be a translational strategy to predict fistula failure and reduce patient morbidity.

## Introduction

Hemodialysis is one of the essential renal replacement therapies for patients with end-stage renal disease^1–3^. Hemodialysis requires patients to have a vascular access such as an arteriovenous fistula (AVF), an artificial vascular graft, or a vascular catheter to allow connection of the dialysis machine to the patient’s bloodstream^4–6^. Among these options, the AVF is the preferred method as AVF are associated with fewer complications and increased durability^7–9^. Nevertheless, the primary patency rate of the AVF is still as poor, only 50–70% at 1 year, with associated interventions and/or re-operations^10–12^. Moreover, 30–60% of AVF require an intervention before clinical use on hemodialysis to assist their maturation^13^. Physiological venous remodeling in the fistula environment, including appropriate neointimal hyperplasia and outward remodeling, is essential for successful AVF maturation^14,15^, but the mechanisms of successful venous remodeling are still poorly understood.

The mouse aortocaval fistula model recapitulates human AVF maturation with venous adaptive remodeling postoperatively through day 28 and then approximately 1/3 of these fistulae fail by day 42, and with greater failure in female mice^16–18^. The mouse AVF model has shown several important processes play a role in venous remodeling, including regulation of markers of arteriovenous differentiation^19–21^, dynamic extracellular matrix production and degradation^22–25^, activation of the innate and adaptive immune responses^26–28^ as well as a role for sex hormones^17,18^. Although these studies have described some of the mechanisms of venous remodeling within the fistula environment, it is still unknown whether these mechanisms predict fistula failure.

High-throughput RNA sequencing (RNA-seq) combined with bioinformatic analyses is a powerful tool to detect changes of global patterns of gene expression using an unbiased approach^29^. We hypothesized that RNA-seq analysis of gene expression during venous remodeling would identify early changes in gene expression that might affect fistula maturation.

## Results

### Quality control and mapping of RNA sequencing data

To assess unbiased transcriptomics data, RNA was extracted from the Inferior vena cava (IVC) of 6 sham-operated mice and 6 mice with patent AVF, with equal numbers of female and male mice on postoperative day 7. The qualities of the 12 total RNA extractions from the 12 IVC samples were sufficiently high with a mean RNA quality number (RQN) 9.0 ± 0.4 (Supplementary Table S1). The raw RNA sequencing data were obtained by preparing messenger RNA (mRNA) libraries and performing next generation sequencing (NGS). The average read counts were 28,364,587 ± 1,700,233 and the sequence lengths were 101 bases in all reads. After quality control, the average read counts were 27,193,479 ± 1,665,134 and the sequence lengths distributed from 36 to 101 bases. Mapping to the reference genome, the average number and percentage of the uniquely mapped reads were 22,392,491 ± 1,456,774 and 82.3 ± 1.6%, respectively. Since the sequencing data were mostly conserved after quality control and appropriately mapped along the reference genome, all mRNA expression values derived from the 12 whole transcriptomics data were utilized.

### Differentially expressed genes (DEG) and gene ontology (GO) enrichment analysis comparing the sham and AVF groups

**Figure 1:**
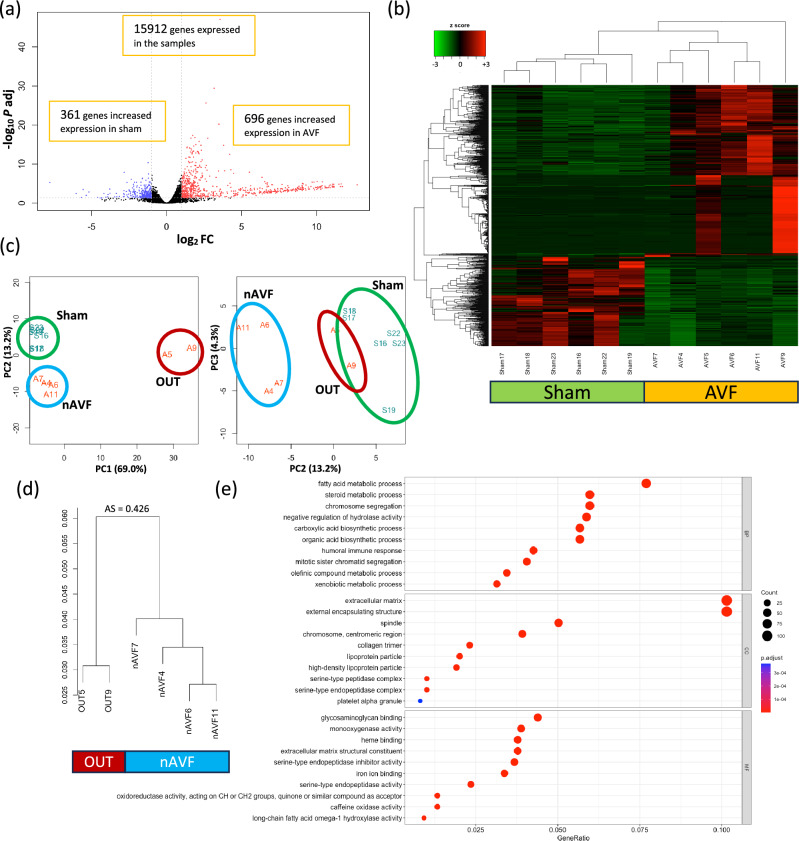
Differentially expressed genes (DEG) and gene ontology (GO) enrichment analyses between the sham and AVF groups. (**a**) Volcano plot of 15,912 genes expressed among the 12 samples (n = 6 sham, n = 6 AVF). 1057 genes were expressed differentially; genes (n = 696) with increased expression in AVF samples are in red dots, and genes (n = 361) with increased expression in sham samples are in blue dots. (**b**) Heatmap plot of the 1057 DEG. (**c**) Principal component analysis (PCA) plot of the 12 samples grouping the 1057 DEG. The 6 sham samples are circled in green; the 4 normal AVF (nAVF) samples are circled in blue; the 2 outlier (OUT) samples are circled in red. (**d**) Dendrogram of the hierarchial sample clustering. The 2 samples in the OUT group were clustered in a group distinct from the other 4 AVF samples (nAVF). The average silhouette (AS) score was 0.426. (**e**) GO enrichment analysis. 317 GO terms were significantly overrepresented among the 1057 DEG. GO terms of the top 10 lowest adjusted P values for each ontology (BP, CC, and MF) are shown. The x-axis shows the ratio of the number of DEG allocated to each GO term compared to the total number of DEG. The color of each dot reflects the adjusted P values. *AVF* arterio-venous fistula, *AS* average silhouette score, *Log2FC* logarithm of a fold change of AVF vs Sham to base 2, *P adj* adjusted P value calculated by the Benjamini–Hochberg procedure, *PC* principal component, *nAVF* normal AVF, *OUT* outliers, *BP* biological process, *CC* cellular component, *MF* molecular function.

To determine differences between the IVC in mice treated with either a sham procedure or an AVF, we identified differentially expressed genes (DEG) from the transcriptomics data. Among the 15,912 genes that were sufficiently expressed in the data set, 1057 genes were expressed differentially between the 2 groups, setting the cutoff as |log2(Fold Change) |≥ 1 and adjusted p value < 0.05. Compared with the IVC in sham mice, 696 genes had significantly increased expression in mice with an AVF, and 361 genes had significantly increased expression in sham-operated mice (Fig. 1a). mRNA expression of the 1057 DEG are shown with a heatmap plot (Fig. 1b) and a principal component analysis (PCA) plot (Fig. 1c) that show homogenous expression of the 6 sham samples, whereas the 6 AVF samples were relatively more heterogeneous. Of note, we observed that the AVF were clustered into 2 groups, a group of 4 AVF samples (Fig. 1c, blue circle) that were significantly different than the 6 sham samples (Fig. 1c, green circle) and a group of 2 AVF samples (Fig. 1c, red circle) that were distinctly different from the 4 AVF samples. The 2 outlying AVF samples (the ‘outliers’ group, OUT) consisted of 1 male (sample #05) and 1 female AVF (sample #09), and the 4 other AVF samples (the ‘normal’ AVF group, nAVF) consisted of 2 female and 2 male AVF. All 6 AVF were patent. To validate the separation of the 2 outlying AVF, a hierarchial sample clustering was performed; the 2 samples in the OUT group were clustered separately from the other 4 samples in the nAVF group, with an average silhouette score 0.426 (Fig. 1d)^30^.

To compare differential gene expression between the sham and AVF groups, gene ontology (GO) enrichment analysis was performed on the 1057 DEG. 317 GO terms were significantly overrepresented among the 1057 DEG. GO terms generally consisted of 3 subgroups, and there were 229 biological process (BP) terms, 20 cellular component (CC) terms and 68 molecular function (MF) terms within the 317 GO terms; the top 10 BP, CC and MF terms with the lowest adjusted p values were shown in Fig. 1e. To validate the heterogeneity within the AVF group, we extracted 13 GO terms related to immunity and 5 GO terms related to coagulation among the 317 GO terms (Supplementary Figs. S1, S2). 64 DEG were included in the 13 immune terms and 37 DEG in the 5 coagulation terms. Heatmap plots of the 64 immune genes and the 37 coagulation genes showed that the 2 AVF samples (sample #05 and #09) forming the OUT group showed different patterns of gene expression than the 4 AVF samples forming the nAVF group (Supplementary Figs. S1, S2). These data suggest that although all 6 of the AVF showed gene expression that was distinct from the sham veins, 2 of the 6 AVF (‘OUT’) showed a different pattern of gene expression compared to 4 of the 6 AVF (‘nAVF’).

### Gene expression microarray analysis comparing sham and AVF groups

To validate the RNA-seq results, we examined our previously performed gene expression microarray analysis^23,31^. In the microarray data, comparing 4 IVC samples from mice with an AVF with 4 IVC samples from sham-operated mice on postoperative day 7, 1196 genes were identified as DEG; MetaCore pathway analysis showed 39 pathways were significantly changed between the sham and AVF (Supplementary Table S2). Among these pathways, many metabolic pathways, such as tricarboxylic acid cycle and mitochondrial long chain fatty acid beta-oxidation, were significantly different. Six pathways related to cell cycle and a pathway of extracellular matrix (ECM) remodeling were also different between the sham and AVF groups. Analysis of several of these pathways showed that each of these metabolic pathways were significantly suppressed in the AVF (Supplementary Table S3). Importantly, almost all these genes downregulated in the AVF in the microarray analysis were also significantly less expressed in the AVF group in the RNA-seq data (Supplementary Table S3). These results show that the RNA-seq data are consistent with the previously reported gene expression microarray data and suggest that at least some metabolic pathways are suppressed during the early phase of venous remodeling.

### Subgroup comparison between nAVF and OUT groups

**Figure 2:**
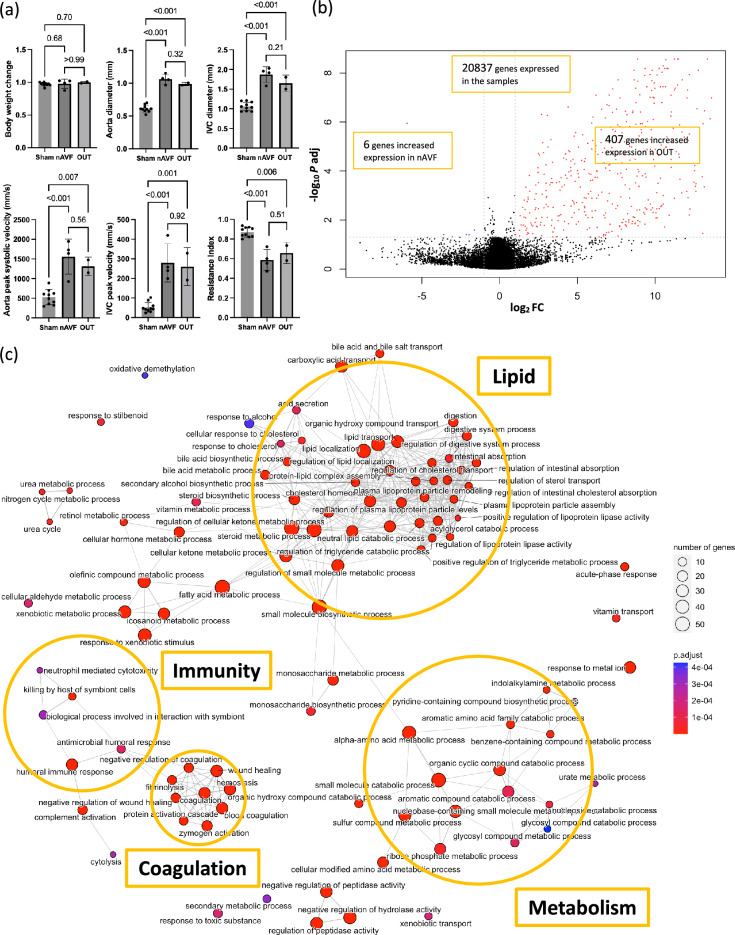
Differentially expressed genes (DEG) and gene ontology (GO) analyses between the normal AVF (nAVF) and outliers (OUT) groups. (**a**) Comparison of ultrasound findings among the sham (n = 6), nAVF (n = 4) and OUT (n = 2) samples. (**b**) Volcano plot of 20,837 genes expressed among the 6 AVF samples. 413 genes were expressed differentially; genes (n = 407) with increased expression in the OUT samples are in red dots, and genes (n = 6) with increased expression in the nAVF samples are in blue dots. (**c**) Enrichment map plot of the top 100 BP terms. Connected edges indicate mutually overlapping DEG sets. *AVF* arterio-venous fistula, *IVC* inferior vena cava, *Log2FC* logarithm of a fold change of nAVF vs OUT to base 2, *P adj* adjusted P value calculated by the Benjamini–Hochberg procedure, *PC* principal component, *BP* biological process, *CC* cellular component, *MF* molecular function.

To further examine the heterogeneity among the AVF group, physiological data were compared (Fig. 2a). There was no significant difference in body weight change (days 0 to 7) among the sham, nAVF and OUT groups. On postoperative day 7, all 6 sham-operated IVC and all 6 AVF were patent. The aorta diameter, IVC diameter, aorta peak systolic velocity, resistance index and IVC peak velocity showed statistically significant increases in both the nAVF and the OUT groups compared to the sham group; however, there were no significant differences between the nAVF and the OUT groups. These data suggest that the nAVF and OUT groups were patent and not physiologically distinguishable at day 7.

DEG between the nAVF and the OUT groups showed that among the 20,837 genes that were sufficiently expressed in the data set, 413 genes were determined as DEG between the 2 groups, setting the cutoff as |log2(Fold Change) |≥ 1 and adjusted p value < 0.05. Compared with the nAVF group, 407 genes had significantly increased expression in the OUT group, whereas only 6 genes had significantly decreased expression (Fig. 2b). The heatmap plot and PCA plot both showed that the 2 groups were well separated with little overlap (Supplementary Fig. S3).

GO enrichment analysis of the 413 DEG between the nAVF and OUT groups showed 239 GO terms consisting of 150 BP terms, 19 CC terms and 70 MF terms. The top 10 BP, CC and MF terms with the lowest adjusted p values are shown in Supplementary Fig. S4. We observed that among the 150 BP terms, many terms related to metabolic processes, such as steroid metabolic process (GO:0008202), fatty acid metabolic process (GO:0006631), α-amino acid metabolic process (GO:1901605), aromatic compound catabolic process (GO:0019439), and glycosyl compound metabolic process (GO:1901657). In addition, terms related to immunity and coagulation were clustered in the enrichment map plots (Fig. 2c, Supplementary Fig. S4).

KEGG pathway enrichment analysis of the 413 DEG between the nAVF group and the OUT group showed 34 KEGG pathways that were significantly overrepresented (Table 1). Similar to the GO enrichment analysis, 16 metabolism-related pathways were detected including carbon metabolism (mmu01200), metabolism of xenobiotics by cytochrome P450 (mmu00980), arachidonic acid metabolism (mmu00590) and biosynthesis of amino acids (mmu01230), consistent with several metabolic processes being active in the OUT group compared with the nAVF group. The complement and coagulation cascades (mmu04610) were also overrepresented with the highest gene ratio and the lowest adjusted p value. These data are consistent with the nAVF and OUT groups having distinct patterns of gene expression, with notable increases in gene expression reflecting increased metabolism, immunity, and coagulation in the OUT group.

**Table 1:** KEGG pathway enrichment analysis comparing the nAVF and the OUT groups.

KEGG ID	Description	Gene ratio	(%)	*P* adj
mmu04610	Complement and coagulation cascades	36/74	48.6	2.80E−29
mmu00140	Steroid hormone biosynthesis	33/69	47.8	1.10E−26
mmu00830	Retinol metabolism	33/76	43.4	3.90E−25
mmu05204	Chemical carcinogenesis—DNA adducts	26/70	37.1	1.20E−17
mmu04976	Bile secretion	26/76	34.2	1.10E−16
mmu00591	Linoleic acid metabolism	16/39	41.0	1.30E−11
mmu04979	Cholesterol metabolism	16/44	36.4	1.10E−10
mmu00983	Drug metabolism—other enzymes	19/76	25.0	1.50E−09
mmu00982	Drug metabolism—cytochrome P450	15/60	25.0	1.50E−07
mmu00053	Ascorbate and aldarate metabolism	10/25	40.0	3.10E−07
mmu00980	Metabolism of xenobiotics by cytochrome P450	14/60	23.3	1.00E−06
mmu00120	Primary bile acid biosynthesis	11/38	47.1	1.60E−06
mmu00260	Glycine, serine and threonine metabolism	8/17	28.9	2.20E−06
mmu01240	Biosynthesis of cofactors	11/38	14.7	4.60E−06
mmu00590	Arachidonic acid metabolism	13/64	20.3	1.30E−05
mmu00040	Pentose and glucuronate interconversions	9/29	31.0	1.30E−05
mmu04975	Fat digestion and absorption	9/30	30.0	1.70E−05
mmu00270	Cysteine and methionine metabolism	11/48	22.9	2.10E−05
mmu00380	Tryptophan metabolism	10/40	25.0	2.50E−05
mmu01230	Biosynthesis of amino acids	12/68	17.6	1.20E−04
mmu04726	Serotonergic synapse	15/107	14.0	1.70E−04
mmu04950	Maturity onset diabetes of the young	6/16	37.5	1.90E−04
mmu05150	Staphylococcus aureus infection	9/42	21.4	2.60E−04
mmu00220	Arginine biosynthesis	6/18	33.3	3.80E−04
mmu00860	Porphyrin metabolism	8/37	21.6	6.20E−04
mmu03320	PPAR signaling pathway	11/70	15.7	6.40E−04
mmu04750	Inflammatory mediator regulation of TRP channels	13/105	12.4	1.80E−03
mmu05207	Chemical carcinogenesis—receptor activation	17/178	9.6	4.70E−03
mmu01200	Carbon metabolism	11/103	10.7	1.70E−02
mmu00430	Taurine and hypotaurine metabolism	4/18	22.2	3.10E−02
mmu02010	ABC transporters	6/42	14.3	3.60E−02
mmu00360	Phenylalanine metabolism	4/19	21.1	3.60E−02
mmu04918	Thyroid hormone synthesis	7/58	12.1	4.60E−02
mmu00340	Histidine metabolism	4/21	19.0	4.90E−02

### Subgroup analysis between the sham and nAVF groups

**Figure 3:**
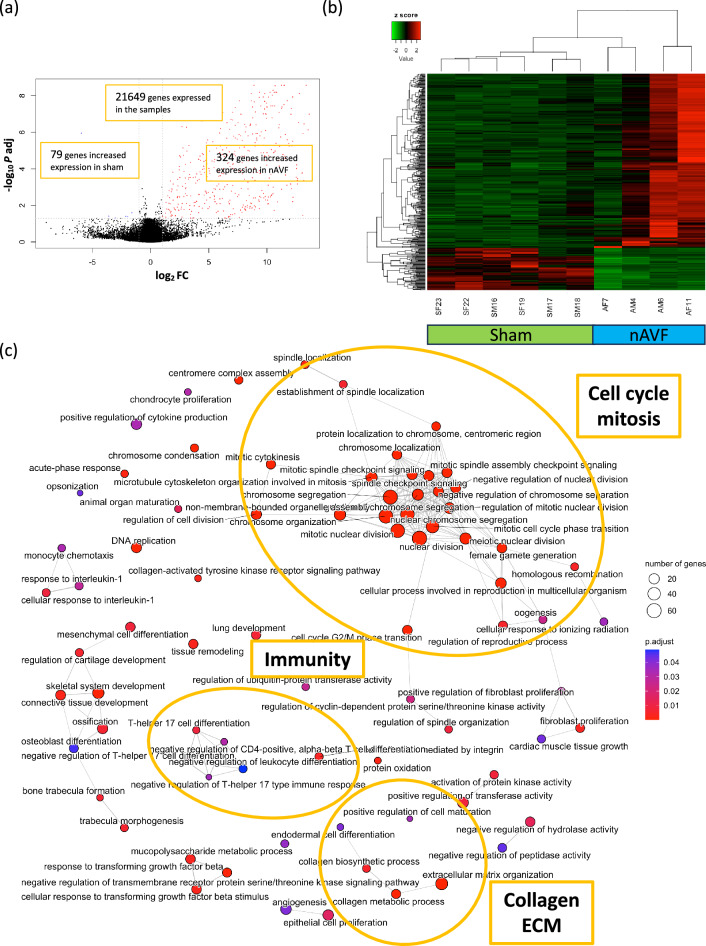
Differentially expressed genes (DEG) and gene ontology (GO) analyses between the sham and normal AVF (nAVF) groups. (**a**) Volcano plot of 21,649 genes expressed among the sham (n = 6) and nAVF (n = 4) samples. 403 genes were expressed differentially; genes (n = 324) with increased expression in the nAVF samples are in red dots, and genes (n = 79) with increased expression in the sham samples are in blue dots. (**b**) Heatmap plot of the 403 DEG. (**c**) Enrichment map plot of the 79 BP terms. Connected edges indicate mutually overlapping DEG sets. *AVF* arterio-venous fistula, *IVC* inferior vena cava, *Log2FC* logarithm of a fold change of nAVF vs Sham to base 2, *P adj* adjusted P value calculated by the Benjamini–Hochberg procedure, *BP* biological process, *CC* cellular component, *MF* molecular function.

To characterize the difference in gene expression between the sham (n = 6) and nAVF (n = 4) groups, we performed another subgroup analysis, without the OUT (n = 2) samples. Among the 21,649 genes expressed sufficiently in the 2 groups, 403 genes had significantly different mRNA expression values, setting the cutoff as |log2(Fold Change) |≥ 1 and adjusted p value < 0.05. Compared with the sham group, 324 genes out of the 403 DEG were significantly upregulated in the nAVF group, while 79 genes were significantly downregulated in the nAVF group (Fig. 3a). The heatmap and PCA plots showed that the 4 samples in the nAVF group were still more heterogeneous compared to the 6 samples in the sham group (Fig. 3b, Supplementary Fig. S5).

GO enrichment analysis of the 403 DEG obtained from the comparison between the sham and nAVF groups identified 122 overrepresented GO terms that consisted of 79 BP terms, 19 CC terms and 24 MF terms. The top 10 BP, CC and MF terms with the lowest adjusted p values are shown in Supplementary Fig. S5. The enrichment map plot of the 79 BP terms showed that terms related to cell cycle and mitosis as well as terms related to immunity and the ECM were all increased in the nAVF group (Fig. 3c). Enrichment map plots of the 19 CC terms and the 24 MF terms showed that cell cycle and extracellular matrix including collagen were increased in the nAVF group (Supplementary Fig. S5). KEGG pathway enrichment analysis of the 403 DEG between the sham and nAVF groups identified 15 KEGG pathways as significantly overrepresented pathways (Table 2), including cell cycle (mmu04110), the p53 signaling pathway (mmu04115), as well as ECM-receptor interaction (mmu04512). These results suggest that the AVF in the nAVF samples showed homogenous expression of genes, at day 7, that could promote venous adaptive remodeling.

**Table 2:** KEGG pathway enrichment analysis comparing the sham and the nAVF groups.

KEGG ID	Description	Gene ratio	(%)	*P* adj
mmu04110	Cell cycle	23/152	15.1	2.27E−09
mmu04974	Protein digestion and absorption	14/76	18.4	1.15E−06
mmu04512	ECM-receptor interaction	14/83	16.9	2.50E−06
mmu04814	Motor proteins	16/171	9.4	5.00E−04
mmu05146	Amoebiasis	11/84	13.1	5.00E−04
mmu04115	p53 signaling pathway	10/69	14.5	5.00E−04
mmu04151	PI3K-Akt signaling pathway	22/310	7.1	8.53E−04
mmu04114	Oocyte meiosis	12/112	10.7	1.19E−03
mmu04510	Focal adhesion	16/196	8.2	1.72E−03
mmu04914	Progesterone-mediated oocyte maturation	10/85	11.8	1.90E−03
mmu05222	Small cell lung cancer	9/90	10.0	1.24E−02
mmu05165	Human papillomavirus infection	19/311	6.1	1.24E−02
mmu04933	AGE-RAGE signaling pathway in diabetic complications	9/99	9.1	2.25E−02
mmu04218	Cellular senescence	12/164	7.3	2.39E−02
mmu04610	Complement and coagulation cascades	6/53	11.3	4.17E−02

### Subgroup analysis between the sham and OUT groups

To further understand the AVF in the OUT group, we compared the sham (n = 6) and OUT (n = 2) groups (Supplementary Fig. S6). Among the 22,163 genes, 701 genes were identified as DEG, setting the cutoff as |log2(Fold Change) |≥ 1 and adjusted p value < 0.05. Compared with the sham group, 667 genes out of 701 DEG were significantly upregulated, whereas 34 genes were significantly downregulated in the OUT group. The heatmap plot and the PCA plot showed that the OUT group was robustly clustered separately from the sham group. GO enrichment analysis of the 701 DEG rendered significantly overrepresented 302 GO terms including 198 BP terms, 25 CC terms and 79 MF terms. The top 10 BP, CC and MF terms with the lowest adjusted p values are shown in Supplementary Fig. S6. As seen in the enrichment map plot, some clusters of metabolic processes were formed. Terms related to coagulation and wound healing were also clustered. Terms of cell cycle and cell division were also distinct in the enrichment map plot of the top 150 BP terms. Although the AVF in the OUT group are distinct from the nAVF, these data suggest that the OUT are nonetheless undergoing adaptive remodeling to the fistula environment, and have increased number of genes regulated in the OUT compared to the nAVF (n = 701 vs. n = 403).

### Integrating the subgroup analyses

To further understand the differences between the nAVF and OUT groups, we merged and reanalyzed the subgroup analysis comparing nAVF with OUT (Fig. 2, Table 1, Supplementary Figs. S3, S4) with the subgroup analysis comparing sham with nAVF (Fig. 3, Table 2, Supplementary Fig. S5) and the subgroup analysis comparing sham with OUT (Supplementary Fig. S6). First, we compared the 2 KEGG pathway analyses (nAVF vs. OUT, Table 1; sham vs. nAVF, Table 2). The complement and coagulation cascade (mmu04610) was the only common pathway that was consistently detected in both analyses. Comparing the OUT group with the nAVF group, F12, F11 and F9 in the intrinsic coagulation pathway were highly expressed in the OUT group (Supplementary Fig. S7). F10, F5, F2 and fibrinogen in the downstream common coagulation pathway were also upregulated in the OUT group. In addition, in the complement cascade, C4BP, C5 and the C6/7/8/9 complex were significantly increased in the OUT group compared to the nAVF group. Comparing the nAVF group with the sham group, no statistical difference was found among coagulation factors as well as among complement factors (Supplementary Fig. S7). Only F8, the most downstream coagulation factor in the intrinsic pathway, was downregulated in the nAVF group. Additionally, serine protease inhibitors (Serpins), which typically suppress the coagulation cascade^32,33^, showed increased expression in the nAVF group compared with the sham group. These data suggest that OUT show increased expression of complement and coagulation factors whereas nAVF do not.

We next integrated the DEG analysis data of the 4 comparisons between AVF and sham, nAVF and sham, OUT and sham, and OUT and nAVF (Table 3). To validate these results, we compared our previously published data directly examining these genes in the identical mouse AVF model. The AVF and nAVF groups showed small but not significant increases in Ephrin B2 and its receptor EphB4, markers of arteriovenous differentiation, consistent with previous reports of their increased expression^19,20,34^. CD44, CD68 and transforming growth factor-β (TGF-β), important immune response markers during the fistula maturation, were significantly increased in the AVF and nAVF groups; their increases on postoperative day 7 after the AVF creation had been reported previously^20,22,23,26^. Genes for collagen type I, type IV, type VIII and type XVIII, tissue inhibitor of metalloproteinase 1 (TIMP1), matrix metalloproteinase 2 (MMP2) and elastin also showed significantly more expression in the AVF and nAVF groups compared to the sham group. Upregulation of these molecules on day 7 were also observed previously^23^. Regarding declining changes, NADH: ubiquinone oxidoreductase (NDUF), vascular endothelial growth factor A (VEGF-A) and programmed death-ligand 1 (PD-L1) were significantly decreased in the AVF group, and these were also previously reported (day 7)^26,28,31^. The OUT group also showed similar changes compared with the sham group. Therefore, these increases and decreases of gene expression in the AVF, nAVF and the OUT groups are consistent with previously published data.

**Table 3:**
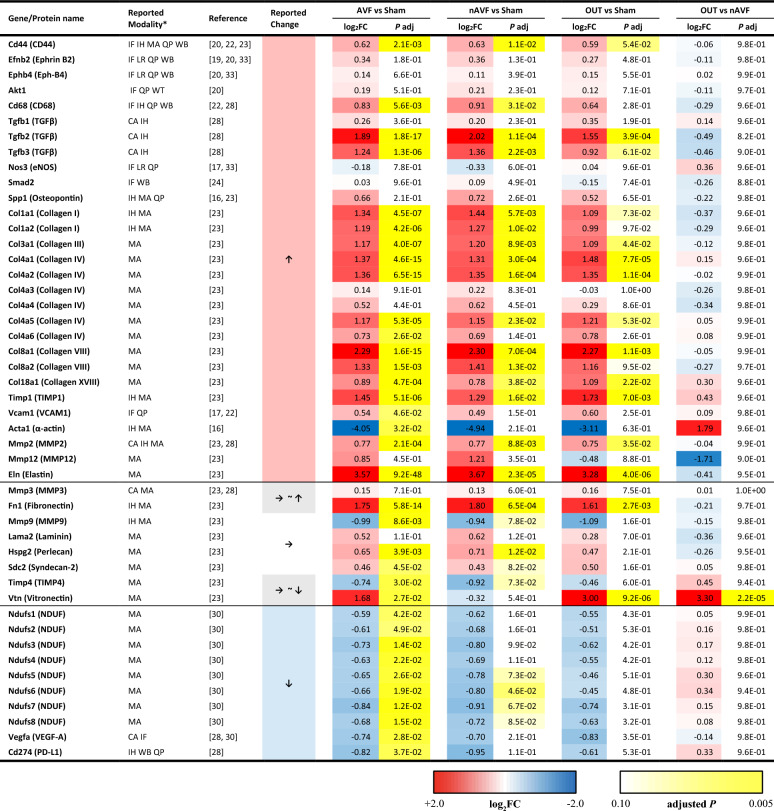
Validation of the next-generation RNA sequencing data with published data using the mouse AVF model.

*CA cytokine array, * F* immunofluorescence, *IH* immunohistochemistry, *LR* literature review, *MA* microarray, *QP* qPCR, *WB* western blotting.

Nevertheless, compared with the nAVF group, the OUT group showed negative log_2_ (Fold Change) in many genes where the AVF and nAVF groups showed increases concordant with the published data (Table 3), such as most of the collagen genes, although no statistical significance was observed. On the other hand, the OUT group, when compared with the nAVF group, showed relatively increased expression of many genes upregulated in the whole AVF group**,** suggesting attenuated regulation of gene expression in the OUT group. In toto, these data suggest that the AVF in the OUT group have a different pattern of adaptive remodeling in the fistula environment compared to both the nAVF group as well as previous published data, e.g., the OUT group are not characterized by the same adaptive venous remodeling as present in the nAVF group.

### Subgroup analysis between female and male AVF

**Figure 4:**
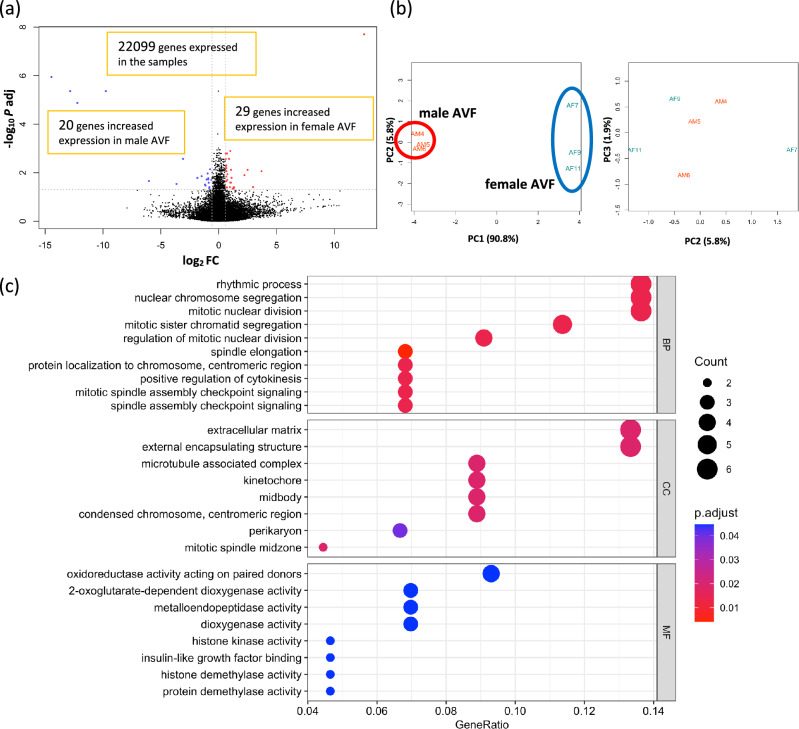
Differentially expressed genes (DEG) and gene ontology (GO) analysis between the female and male AVF. (**a**) Volcano plot of 22,099 genes expressed among the female (n = 3) and male (n = 3) AVF samples. 49 genes were expressed differentially; genes (n = 29) with increased expression in the female AVF samples are in red dots, and genes (n = 20) with increased expression in the male AVF samples are in blue dots. (**b**) Principal component analysis (PCA) plot of the 6 AVF samples grouping the 49 DEG. The 3 female AVF samples are circled in blue; the 3 male AVF samples are circled in red. (**c**) GO enrichment analysis. 63 GO terms were significantly overrepresented among the 49 DEG. *AVF* arterio-venous fistula, *Log2FC* logarithm of a fold change of the female AVF vs the male AVF to base 2, *P adj* adjusted P value calculated by the Benjamini–Hochberg procedure, *PC* principal component, *BP* biological process, *CC* cellular component, *MF* molecular function.

To determine whether sex differences in gene expression were present in the AVF group, we performed a subgroup analysis between the female AVF samples (n = 3) and the male AVF samples (n = 3). With the cutoff as |log2(Fold Change) |≥ 1 and adjusted *p* value < 0.05, only 24 DEG consisting of 12 upregulated genes and 12 downregulated genes were identified. To avoid missing important differences in gene expression, we lowered the threshold level for the fold change from 2.0 times to 1.5 times, that is |log2(Fold Change) |≥ 0.585. Even with this threshold change, only 49 genes were differentially expressed, with 29 genes upregulated, and 20 genes downregulated in the female AVF (Fig. 4a). As expected, 2 genes out of the 29 genes expressed greater in the female AVF were present on the X chromosome, and 5 of the 20 genes less expressed in the female AVF were present on the Y chromosome (Supplementary Table S4). The heatmap plot and PCA plot of the 49 DEG showed that no prominent homogeneity was observed in either group (Fig. 4b, Supplementary Fig. S8). GO enrichment analysis of the 49 DEG showed that 63 GO terms were statistically significant, with 47 terms belonging to BP, 8 terms belonging to CC and 8 terms belonging to MF. Among the top 10 BP terms with the lowest adjusted *p* values, 8 CC terms and 8 MF terms are shown in Fig. 4c. Enrichment map plots of the 3 ontology subgroups are shown in Supplementary Fig. S8. These data are consistent with very few differences in gene expression detectable between patent female and male AVF on postoperative day 7.

## Discussion

Our data shows heterogeneous patterns of mRNA expression on postoperative day 7 among the AVF group compared to the relatively homogenous sham-operated group (Fig. 1b,c Supplementary Figs. S1, S2), although many genes related to metabolism were significantly downregulated in the AVF group, which is consistent with previous microarray data (Supplementary Table S3). Interestingly, the AVF group was divisible into two distinct subgroups, nAVF and OUT (Fig. 1c,d), with different patterns of expression including coagulation, immunity, lipid, and many metabolic processes (Fig. 2c, Table 1, Supplementary Fig. S4), despite all AVF among both groups being patent and physiologically indistinguishable (Fig. 2a). The nAVF group showed overrepresentation in immunity, extracellular matrix including collagens, and cell cycle (Fig. 3c, Table 2, Supplementary Fig. S5) compared to sham. The OUT group showed overrepresentation in cell cycle and metabolic processes, but also showed distinct activation of the coagulation and complement systems (Supplementary Fig. S7). Previously published data was used to validate these findings (Table 3). These data suggest that the AVF group, including the nAVF and OUT groups, have an expression profile compatible with adaptive remodeling, whereas a significant minority of AVF (OUT) may be predisposed to fistula failure with additional activation of genes in the metabolism, coagulation and inflammation pathways.

Our data shows that a significant minority of the AVF (2 of 6 samples) are associated with different patterns of mRNA expression from the majority (4 of 6) of AVF (Fig. 1b–d). We have previously reported, using the same murine AVF model, approximately a third of the patent AVF gradually fail to remain patent beginning at day 21 through day 42^16,35^. These similar data suggests that our mouse AVF model shows a rate of failure to mature that corresponds to that seen with human fistulae. Although the clinical outcome of AVF remains inadequate for many patients, biological mechanisms that regulate AVF maturation and failure are still poorly understood^36^. Patient characteristics such as age, female sex and diabetes as well as physiological parameters such as the preoperative venous diameter and the postoperative fistula flow velocity may be related to fistula failure^37–39^, but these clinical markers have not led to rigorous molecular or physiological biomarkers that correlate with failure. One potential biomarker, low serum ratio of matrix metalloproteinase/tissue inhibitor of metalloproteinase (MMP/TIMP) at the time of fistula surgery was associated with subsequent maturation failure in humans^40^. In our data, the OUT group showed relatively low MMP12 expression and high TIMP4 expression compared to the nAVF group as well (Table 3). Moreover, the coagulation cascade in the OUT group was upregulated (Fig. 2c, Supplementary Fig. S7). Activated coagulation and thrombosis are clinically related to AVF failure^41^. In addition, genotype polymorphisms of factor V, prothrombin (factor II) and plasminogen activator inhibitor I (PAI-I) were also associated with AVF failure in humans^42,43^. In a randomized controlled trial, the combination of oral prostacyclin analog and clopidogrel improved early AVF patency and maturation compared to placebo^44^. These similarities suggest the OUT group may be destined to fail to achieve adaptive remodeling.

Interestingly, although metabolism was generally suppressed in the AVF samples compared to the sham (Supplementary Table S3), many metabolic processes were relatively less downregulated in the OUT group when compared to the nAVF group (Fig. 2c, Supplementary Fig. S4). However, the relationship between AVF maturation and metabolism has been poorly documented. Using a microarray, we previously reported that oxidative phosphorylation, mitochondrial long chain fatty acid β-oxidation and mitochondrial unsaturated fatty acid β-oxidation were significantly downregulated in the mouse AVF model on day 7 compared to sham veins^31^. Endothelial cells prefer anaerobic glycolytic metabolism to oxidative phosphorylation to produce ATP^45,46^, perhaps to minimize the effects of reactive oxygen species and oxidative stress^47^. Therefore, it is possible that suppression of several metabolic processes could be a key signature of successful adaptive remodeling and ultimately AVF maturation.

In contrast to the OUT group, the nAVF group may represent physiological adaptive venous remodeling. The nAVF group showed significant changes in ECM and immunity as well as cell cycling (Fig. 3c, Table 3, Supplementary Fig. S5), compared to the sham group. The regulation of AVF maturation is complex and remains incompletely understood. Immediately after AVF creation, the vein accommodates to a new fistula environment characterized by arterial inflow without peripheral vascular resistance, and thus the vein is exposed to high flow, high pressure, high shear stress, and high oxygen tension^48–50^ to which the vein undergoes adaptive outward remodeling and wall thickening^15,51–53^. The adaptative response involves not only cell proliferation but also a constellation of biological processes including ECM remodeling^23,47,51^. Degradation of ECM by the upregulation of the MMP family plays a key role in the early maturation phase in order for the vein to remodel. Likewise, proper expression of TIMP, collagens, elastins, and other structural proteins is essential to regulate the degradation and synthesis of ECM^23,54^. Moreover, as the nAVF group showed overrepresentation of immunity, immune response including both innate and adaptive systems such as accumulation of macrophages and T cells is also important for the AVF maturation^24,26,28,55^. Our data shows heterogeneous responses in the group of veins, suggesting that patterns of gene expression predict success or failure of venous adaptive remodeling.

Although RNA-seq is becoming a popular biological technique, few studies have reported use of RNA-seq to study AVF maturation. In a study that examined paired vein and AVF samples in humans^56^, 102 DEG were identified in association with subsequent AVF maturation failure. Among these DEG, proteoglycan-modifying enzymes (HYAL2 and HPSE) and metalloproteinases (MMP9, MMP19, ADAMTS9, and ADAMTS14) showed decreased expression in failure cases. However, no upregulation of metabolic process was reported in the study. The timing of specimen collection in this study was 2–4 months after AVF creation, so active changes of metabolic signatures in failure cases might be difficult to detect.

Our study surprisingly found that comparison between female and male AVF showed almost insignificant differences (Fig. 4, Supplementary Table S4, Supplementary Fig. S8), although female sex is a well-known risk factor of AVF maturation failure in humans^7,57^. Moreover, our murine AVF model showed significantly worse patency rate in female mice beginning day 21 up to day 42^17^. We also reported gonadectomy in female mice diminished accumulation of monocytes, macrophages, and T cells in the venous wall on day 3 and day 7, suggesting female sex hormones regulate AVF maturation via immune cell recruitment^18^. Similarly, RNA-seq and bioinformatics analyses of human AVF samples showed that activated γδ-T cells and mast cells were observed only in women^58^. Since our RNA-seq data does not show sex differences that emerge later than day 7 in this model, female sex hormones may have later influence on the immune cell response to venous adaptive remodeling.

In conclusion, RNA-seq showed significantly heterogeneous mRNA expression on day 7 in the murine aortocaval AVF model. Despite physiologically indistinctive features of the AVF, a small subset of AVF undergoes not only normal remodeling, but also several biological processes including diminished ECM remodeling, activated coagulation and inflammation as well as relatively upregulated metabolism, that suggest predisposition to maladaptive remodeling that could lead to fistula failure. These data suggest that detection of these processes, such as metabolism, might be a viable translational strategy to predict fistula failure and reduce patient morbidity.

## Materials and methods

### Arteriovenous Fistula (AVF) model

All animal experiments were performed in compliance with federal guidelines and the ARRIVE guidelines and with approval from the Institutional Animal Care and Use Committee of Yale University.

10-week-old wild-type female and male C57BL/6 J mice were purchased from Jackson Laboratory (Bar Harbor, ME, USA). Infrarenal aortocaval fistulae were created as previously described^16,35^. Briefly, inhaled 2–2.5% isoflurane in 1.0 L/min 0_2_ was used for general anesthesia, and extended-release buprenorphine (Ethiqa XR; North Brunswick, NJ, USA) was administered subcutaneously with a dosage of 3.25 mg/kg body weight for intraoperative and postoperative analgesia. The abdominal aorta and the IVC were exposed with a midline laparotomy; the bowels were gently retracted. After the vessels were clamped at an immediately caudal level of the origins of the left renal artery and vein, a fistula between the vessels was created by using a 25-gauge needle to puncture the aorta and IVC, from a left lateral side of the aorta into a lumen of the IVC at a level cranial to the aortic bifurcation. Hemostasis of the puncture site on the lateral aortic wall was achieved by a gentle compression with a surrounding retroperitoneal tissue. A pulsatile bright-red blood flow visible intraoperatively through the IVC wall was considered as a primary technical success of the AVF creation. Sham-operated mice underwent all the same steps except the needle puncture.

### Ultrasound measurements

A Vevo 770 High-Resolution Imaging system (FUJIFILM Visual Sonics, Toronto, Canada) with an RMV704 transducer with a center frequency of 40 MHz was utilized for a sonographic assessment just before the operation (day 0) and on postoperative day 7 as described previously^16,35^. 10 sham-operated mice (5 female and 5 male animals) and 6 mice with a AVF (3 female and 3 male animals) were assessed. Briefly, under general anesthesia with inhaled 1.5% isoflurane in 1.0 L/min O_2_, the abdominal aorta and the IVC were identified at a level of left renal artery and vein. Diameters of the vessels were measured at an immediately caudal level of the origins of the left renal artery and vein in a transverse view under B-mode. Velocities of blood flow in the vessels were also measured at the same level in a longitudinal view under pulse-wave mode. An AVF was considered patent when increased diameters and velocities of the vessels were observed on postoperative day 7. Resistance index was calculated by (aorta peak systolic velocity – aorta end-diastolic velocity) / aorta peak systolic velocity.

### RNA extraction and RNA sequencing

For poly-A mRNA sequencing, IVC from 6 sham-operated mice and 6 mice with a AVF were collected. Among these animals, the 6 AVF were from the same mice used for the ultrasound assessment above. Euthanasia method for all animals was exsanguination under anesthesia with inhaled 3–4% isoflurane in 1.0 L/min O_2_, consistent with recommendations of the American Veterinary Medical Association Guidelines on Euthanasia and complying with local Veterinary Clinical Services policies. All IVC samples were harvested after circulatory flushing with normal saline on postoperative day 7. The range of the extracted IVC from mice with an AVF was from just above the fistula opening through just below the origin of the left renal vein. The tissues were immediately submerged in RNAprotect Tissue Reagent (QIAGEN, Hilden, Germany) and incubated overnight at 4 °C, then stored at − 20 °C without the reagent. Total RNA was extracted using RNeasy Mini Kit with DNase I (QIAGEN, Hilden, Germany) according to the manufacturer’s protocol. Total RNA quality and concentration were estimated with A260/A280 and A260/A230 ratios measured using a NanoDrop spectrophotometer (Thermo Scientific, Wilmington, DE, USA).

Preparation and sequencing of poly-A mRNA sequencing libraries were performed by the Yale Center for Genome Analysis (YCGA). RNA integrity was estimated using an Agilent 2100 Bioanalyzer (Agilent Technologies, Santa Clara, CA, USA). Samples with RNA integrity number (RIN) values of ≥ 8.0 were used for analysis. Poly-A mRNA was purified from approximately 200 ng of total RNA and the stranded mRNA sequencing libraries were constructed using KAPA mRNA HyperPrep Kit for Illumina sequencing (Roche, Basel, Switzerland). Indexed libraries were quantified by qRT-PCR using KAPA Library Quantification Kits (Roche, Basal, Switzerland) and insert size distribution was determined using an Agilent 2100 Bioanalyzer. Samples with a yield of ≥ 0.5 ng/µL and a size distribution of 150–300 bp were sequenced. Sample concentrations were normalized to 1.2 nM and loaded onto an Illumina NovaSeq flow cell (Illumina, San Diego, CA, USA) at a concentration that yielded 25 million passing filter clusters per sample. Samples were sequenced using 101 bp paired-end sequencing on an Illumina NovaSeq 6000 (Illumina, San Diego, CA, USA). Data generated during sequencing runs were simultaneously transferred to the YCGA high-performance computing cluster.

### Differential expression analysis and enrichment analysis

The FASTQ files of raw sequencing reads were imported into a computing cluster based on Red Hat Linux computing system and analyzed. For the purpose of quality control, poly A and poly T sequences, Illumina adaptor sequences and low-quality sequences in the reads were deleted using PRINSEQ v0.20.4 and Trimmomatic v0.39 software^59,60^. The trimmed sequencing data were aligned to the GRCm39 reference genome with the gene annotation information (Ensembl release 106)^61^ and expression values for each gene were counted using STAR v2.7.7a and RSEM v1.3.3 software^62^ (Supplementary Code S1).

Further analyses were performed using packages in R v4.2.3 and RStudio v2023.03.0 build 386 on the macOS computing system. Differentially expressed genes (DEG) were identified from the dataset using the edgeR v3.40.2 package^63^ (Supplementary Code S2). To identify DEG and perform post-hoc analyses among subgroups, we used the TCC v1.38.0 and baySeq v2.32.0 packages^64–66^ (Supplementary Code S3, S4, S5). To exclude low-expressed genes, only genes with total counts per million (CPM) greater than 1 in at least 2 samples were used for the analysis between the sham and the AVF groups (Supplementary Code S2). For subsequent subgroup analyses, only genes with total expression counts greater than 12 throughout the 12 samples were used (Supplementary Code S3, S4, S5). Gene Ontology (GO) enrichment analysis and Kyoto Encyclopedia of Genes and Genomes (KEGG) pathway enrichment analysis were carried out using the clusterProfiler v4.6.2 package^67^ (Supplementary Code S2, S3, S4, S5). Visualization of the enrichment analyses were rendered by the clusterProfiler and the pathview v1.38.0 packages^68^.

### Gene expression microarray analysis

To validate the RNA-seq data, we referred to microarray data that was previously described^23,31^. Briefly, the IVC of sham-operated mice or mice with an AVF were collected on postoperative day 7 (n = 4). Total RNA was isolated from each sample, and 200 ng of isolated RNA was used for whole genome gene expression microarray analysis using the Mouse Gene 1.0 ST array (Affymetrix, Santa Clara, CA, USA). DEG were identified with a false discovery rate < 0.05 and an absolute fold change > 2.0. Pathway enrichment analysis was performed using MetaCore (Thomson Reuters, New York, NY, USA).

### Statistical analysis

Statistical analyses of the sequencing data were performed with the above-mentioned packages in R, where an adjusted P value of < 0.05 and an absolute fold change of ≥ 2.0 were considered statistically significant to identify DEG unless otherwise specified. Adjusted P values of < 0.05 were considered statistically significant including for the GO and KEGG enrichment analyses. The adjusted P values were determined by applying the Benjamini–Hochberg multiplicity correction method^69^.

All other statistical analyses were performed with GraphPad Prism 9 v9.4.1 (GraphPad Software, San Diego, CA, USA). Data are represented as mean ± standard error of the mean. For multiple group comparison, the Shapiro–Wilk test was used to determine normality of the distributions; for normally distributed data, one-way ANOVA followed by Tukey’s post hoc test was used whereas for data without a normal distribution, the Kruskal–Wallis test followed by the Dunn post hoc test was performed. Statistical significance was determined as a P value < 0.05.

### Supplementary Information

Supplementary Information.

## Data Availability

All custom scripts are available as Supplementary Code S1–S5. The raw RNA sequencing data and the processed data generated in this study have been submitted to the NCBI Gene Expression Omnibus (GEO) database under accession number GSE244317.
