# Supplementary material for: Heterogeneous gene expression during early arteriovenous fistula remodeling suggests that downregulation of metabolism predicts adaptive venous remodeling
Source: Sci Rep. 2024 Jun 10;14:13287. doi: 10.1038/s41598-024-64075-8 (PMC11164895; doi:10.1038/s41598-024-64075-8)
Supplement: Supplementary file 1 — Supplementary Information. [file 41598_2024_64075_MOESM1_ESM.pdf]

Supplemental Data

Title

Heterogeneous gene expression during early arteriovenous fistula remodeling suggests that downregulation of metabolism predicts adaptive venous remodeling

Authors

Yuichi Ohashi, MD[1,2,3], Clinton D. Protack, MD, PhD,[1,2] Yukihiko Aoyagi, MD, PhD[1,2,4], Luis Gonzalez, PhD[1,2], Carly Thaxton, MD[1,2], Weichang Zhang, MD, PhD[1,2], Masaki Kano, MD, PhD[1,2,5], Hualong Bai, MD, PhD[1,2], Bogdan Yatsula, PhD[1,2], Rafael Alves, BM[1,2], Katsuyuki Hoshina, MD, PhD[3], Eric B Schneider PhD[6], Xiaochun Long PhD[7], Rachel J. Perry, PhD[8,9], Alan Dardik, MD, PhD[1,2,9,10, *]

Affiliations

[1] Vascular Biology and Therapeutics Program, Yale School of Medicine, New Haven, Connecticut, United States

[2] Department of Surgery, Yale School of Medicine, New Haven, Connecticut, United States

[3] Division of Vascular Surgery, Department of Surgery, The University of Tokyo, Tokyo, Japan

[4] Department of Surgery and Science, Graduate School of Medical Sciences, Kyushu University, Fukuoka, Japan

[5] Department of Cardiovascular Surgery, Tokyo Medical University, Tokyo, Japan

[6] Vascular Biology Center, Medical College of Georgia at Augusta University, Augusta, Georgia, United States

[6] Center for Health Services and Outcomes Research, Department of Surgery, Yale School of Medicine, New Haven, Connecticut, United States

[8] Department of Internal Medicine, Yale School of Medicine, New Haven, Connecticut, United States

[9] Department of Cellular and Molecular Physiology, Yale School of Medicine, New Haven, Connecticut, United States

[10] Department of Surgery, Veterans Affairs Connecticut Healthcare System, West Haven, Connecticut, United States

***Correspondence**: Alan Dardik, Yale School of Medicine, 10 Amistad Street, Room 437, PO Box 208089, New Haven, Connecticut 06520-8089 United States. Tel: 203-737-2082. Email: alan.dardik@yale.edu

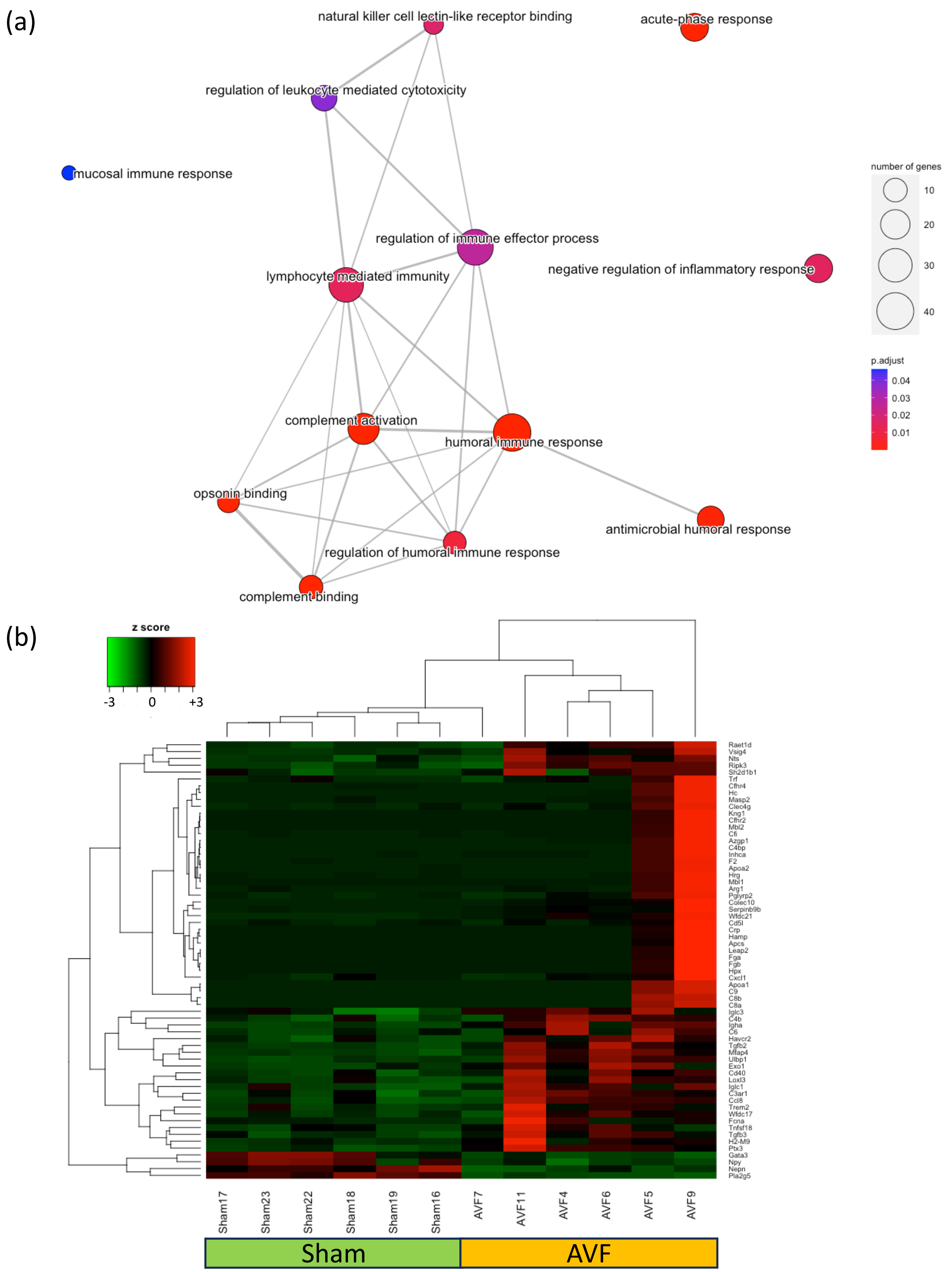

Supplementary Figure S1: Difference in the Immune system comparing the sham and AVF groups.

(a) Enrichment map plot of the 13 GO terms related to the immune system. Connected edges indicate mutually overlapping DEG sets. (b) Heatmap plot of the 64 immune genes included in the 13 GO terms.

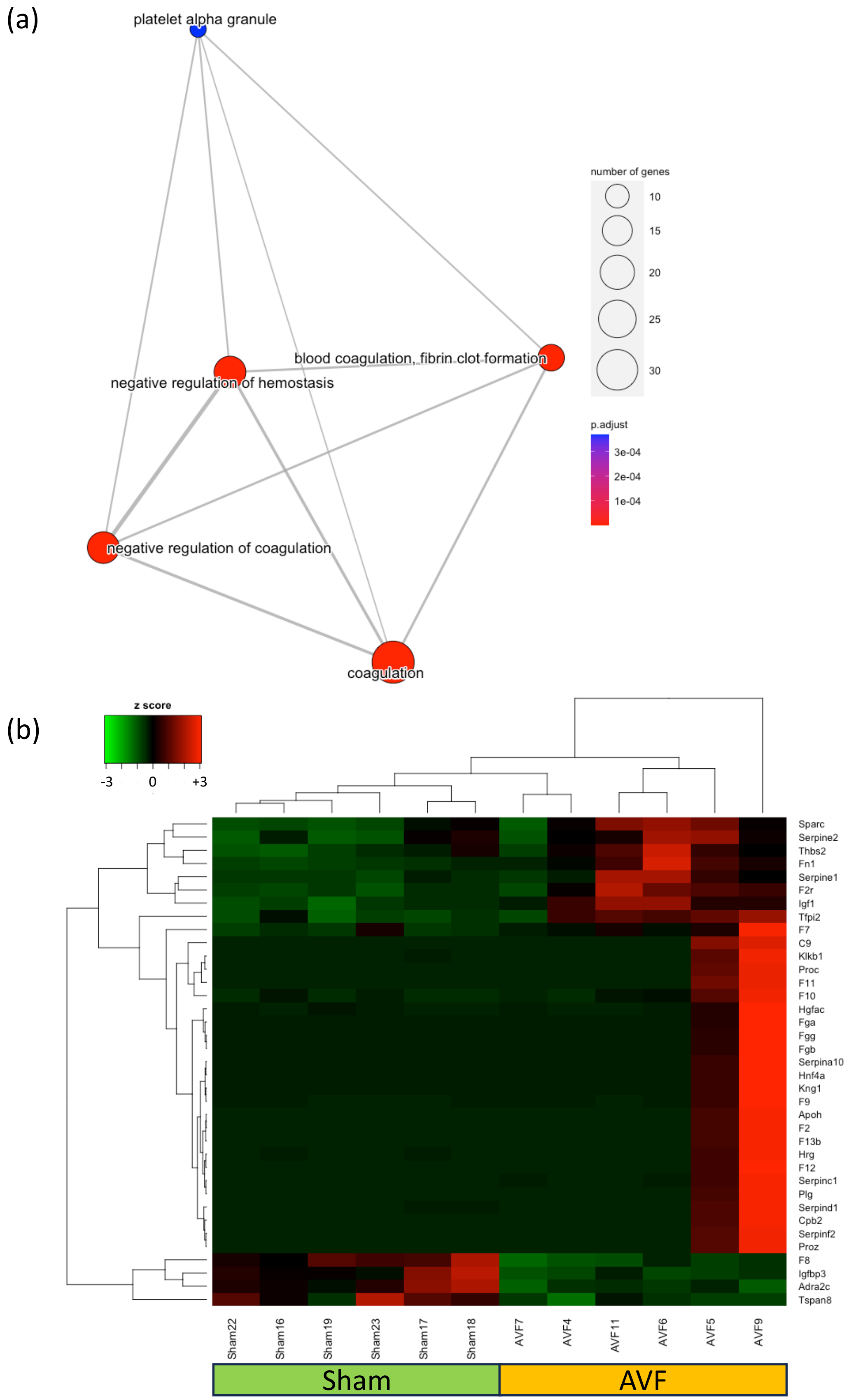

Supplementary Figure S2: Difference in the coagulation comparing the sham and AVF groups.

(a) Enrichment map plot of the 5 GO terms related to the coagulation cascade. Connected edges indicate mutually overlapping DEG sets. **(b)** Heatmap plot of the 37 DEG included in the 5 GO terms related to the coagulation cascade.

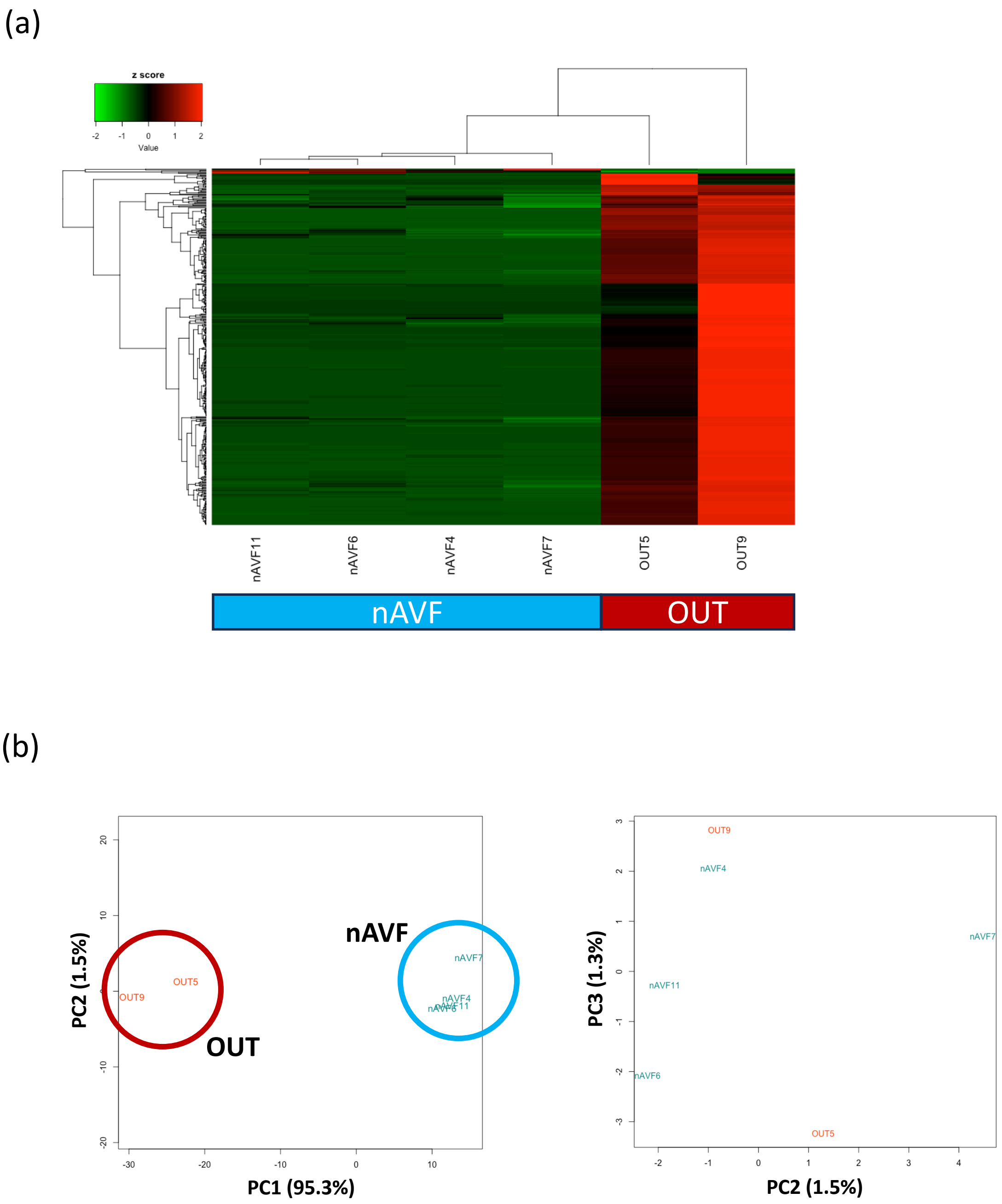

Supplementary Figure S3: Differentially expressed genes (DEG) between the normal AVF (nAVF) and outliers (OUT) groups.
(a) Heatmap plot of the 413 DEG. **(b)** Principal component analysis (PCA) plot of the 6 AVF samples grouping the 413 DEG. The 4 normal AVF (nAVF) samples are circled in blue; the 2 outlier (OUT) samples are circled in red.

(a)

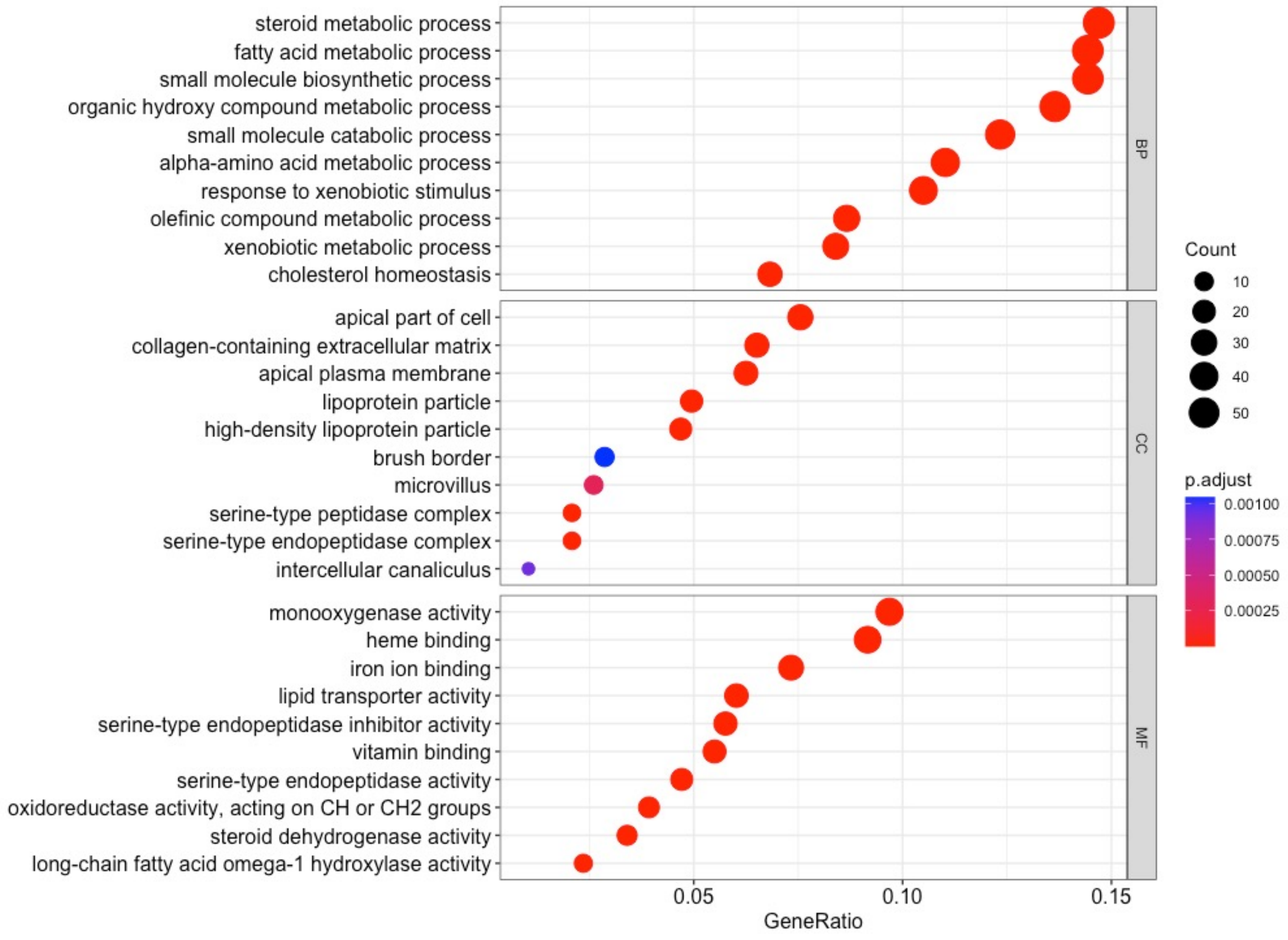

Supplementary Figure S4: Enrichment analysis comparing the normal AVF (nAVF) and outliers (OUT) groups.
(a) GO enrichment analysis. 239 GO terms (150 BP, 19 CC, and 70 MF) were significantly overrepresented among the 413 DEG. GO terms with the top 10 lowest adjusted P values for each ontology (BP, CC, and MF) are shown. ***(Continues)***

(b)

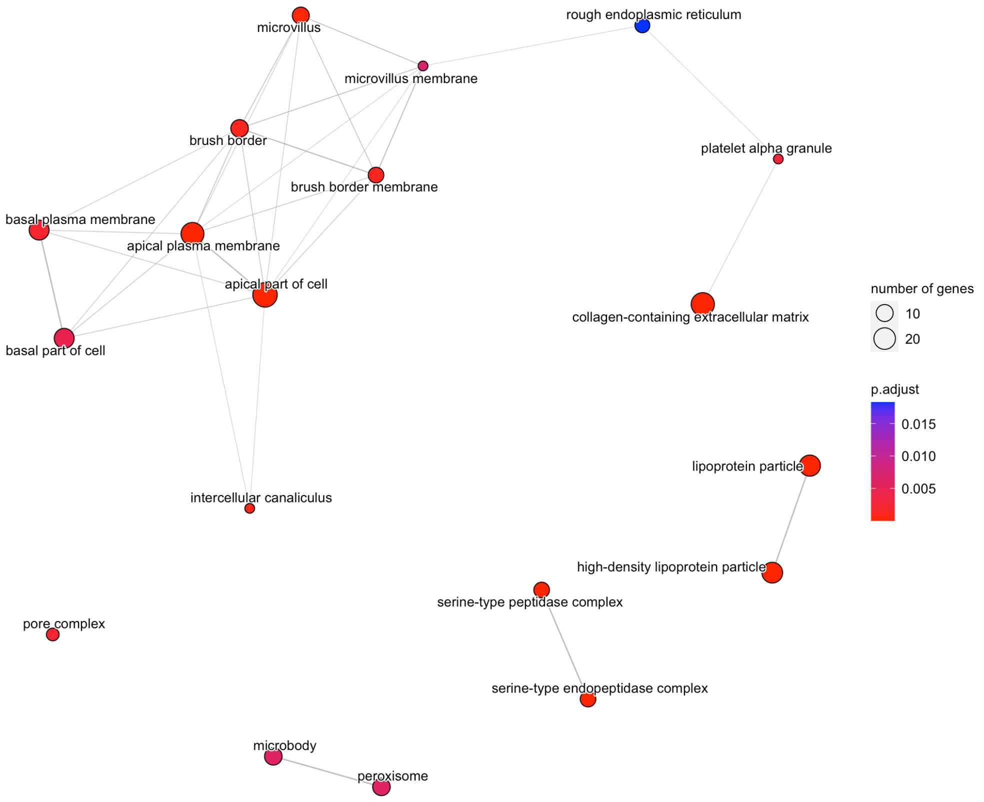

(c)

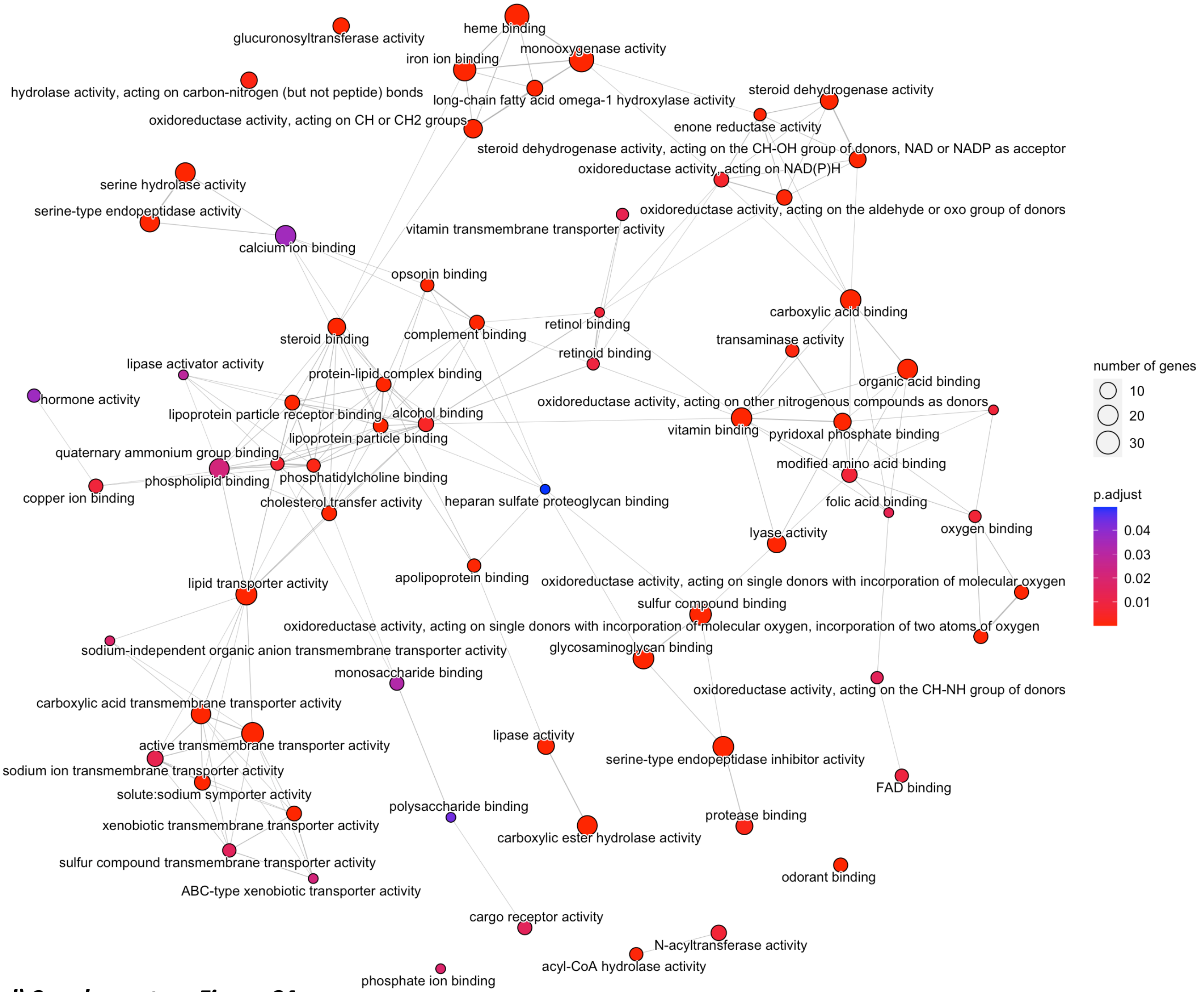

(Continued) Supplementary Figure S4.

(b) Enrichment map plot of the 19 CC terms. **(c)** Enrichment map plot of the 70 MF terms.

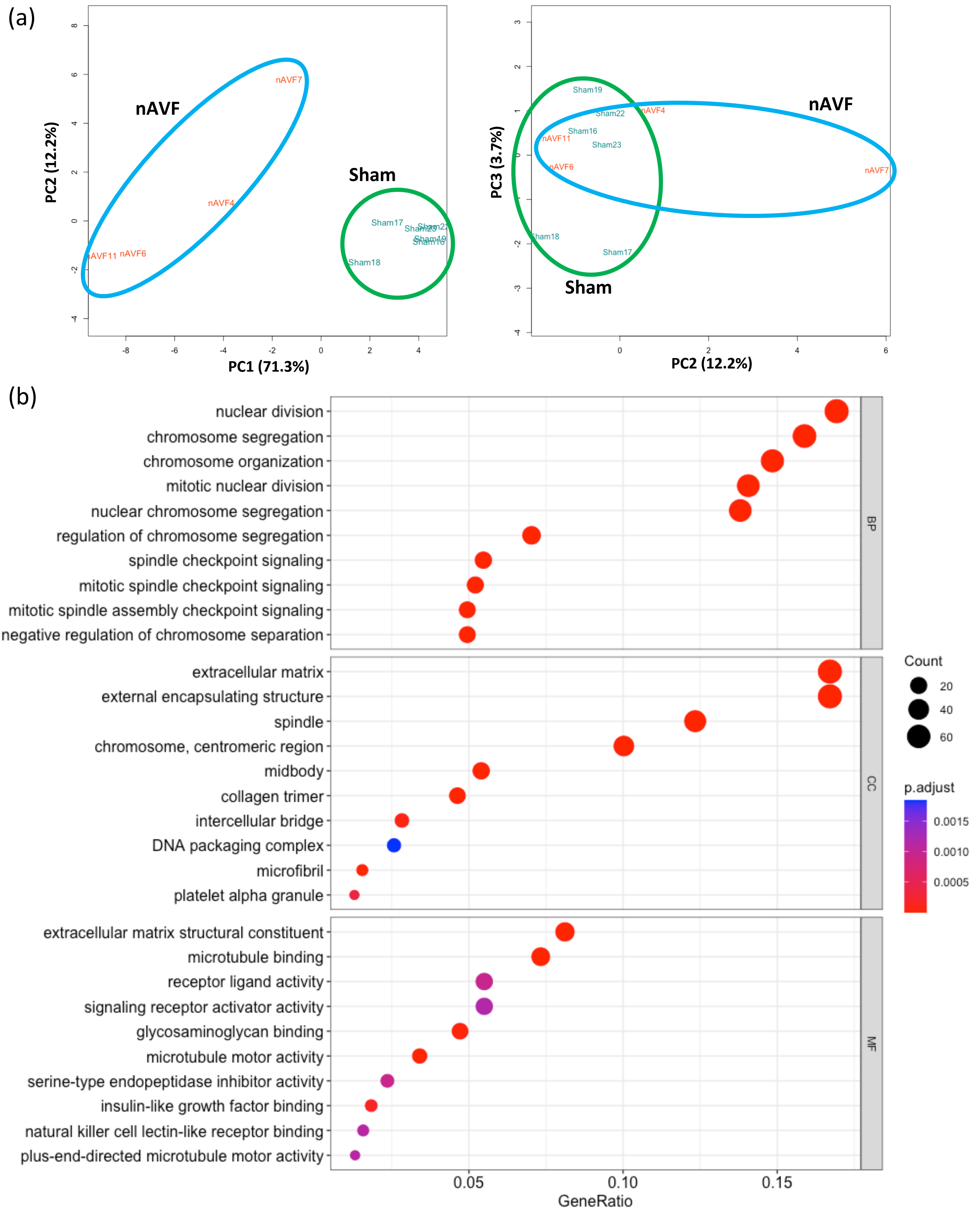

Supplementary Figure S5: Comparison between the sham and normal AVF (nAVF) groups.

(a) Principal component analysis (PCA) plot of the 10 samples (n=6 sham, n=4 nAVF) grouping the 403 DEG. The 6 sham samples are circled in green; the 4 nAVF samples are circled in blue. **(b)** GO enrichment analysis. 122 GO terms (79 BP, 19 CC, and 24 MF) were significantly overrepresented among the 403 DEG. GO terms with the top 10 lowest adjusted P values for each ontology (BP, CC, and MF) are shown. ***(Continues)***

(c)

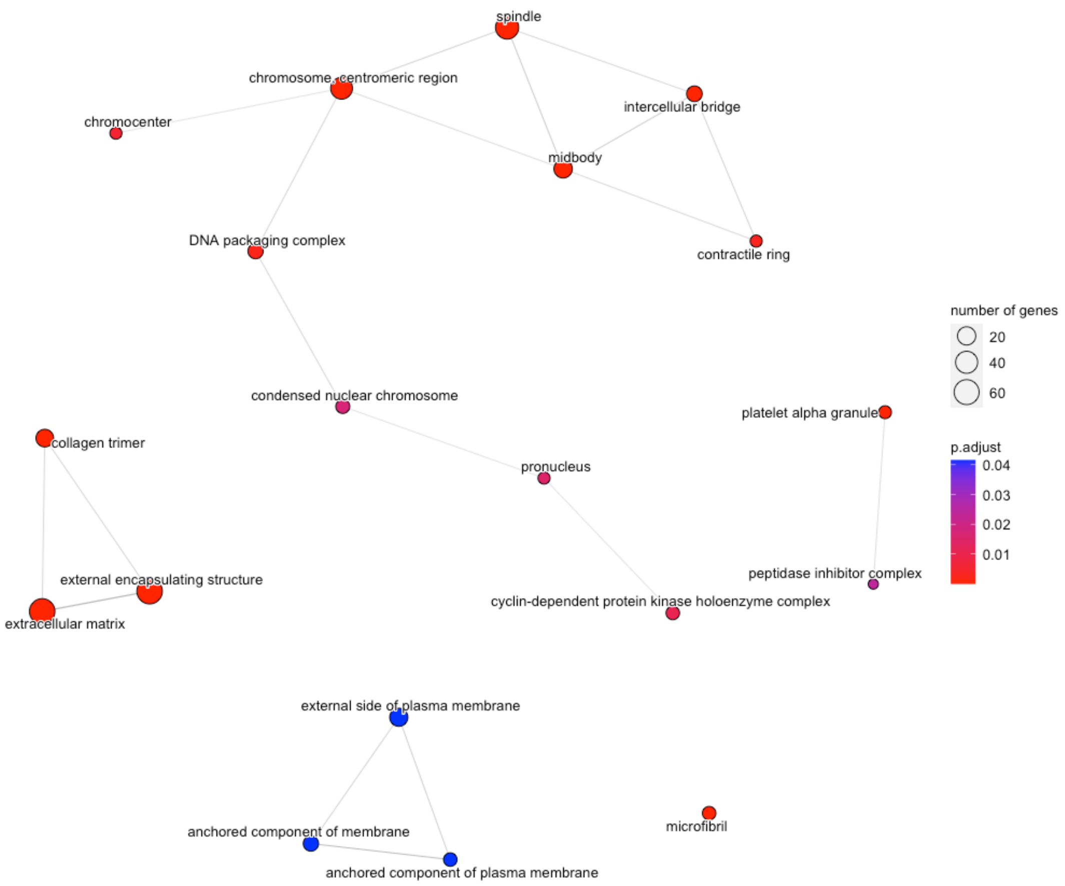

(d)

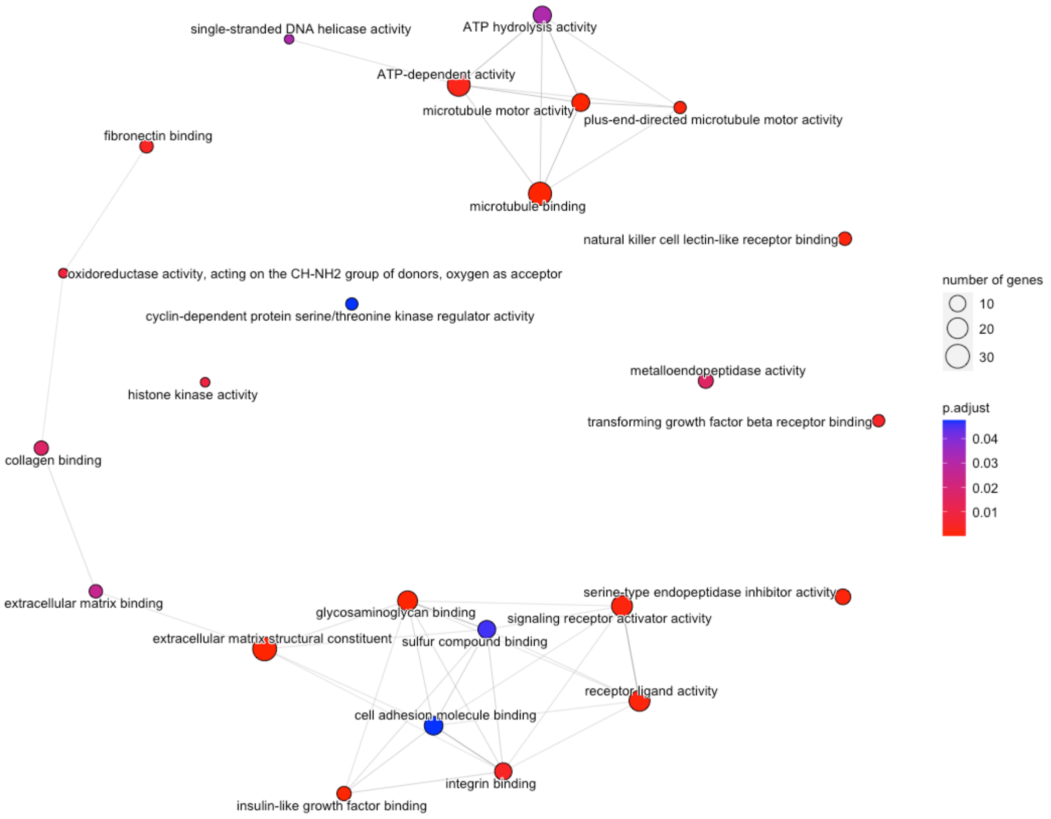

(Continued) Supplementary Figure S5.

(c) Enrichment map plot of the 19 CC terms. **(d)** Enrichment map plot of the 24 MF terms.

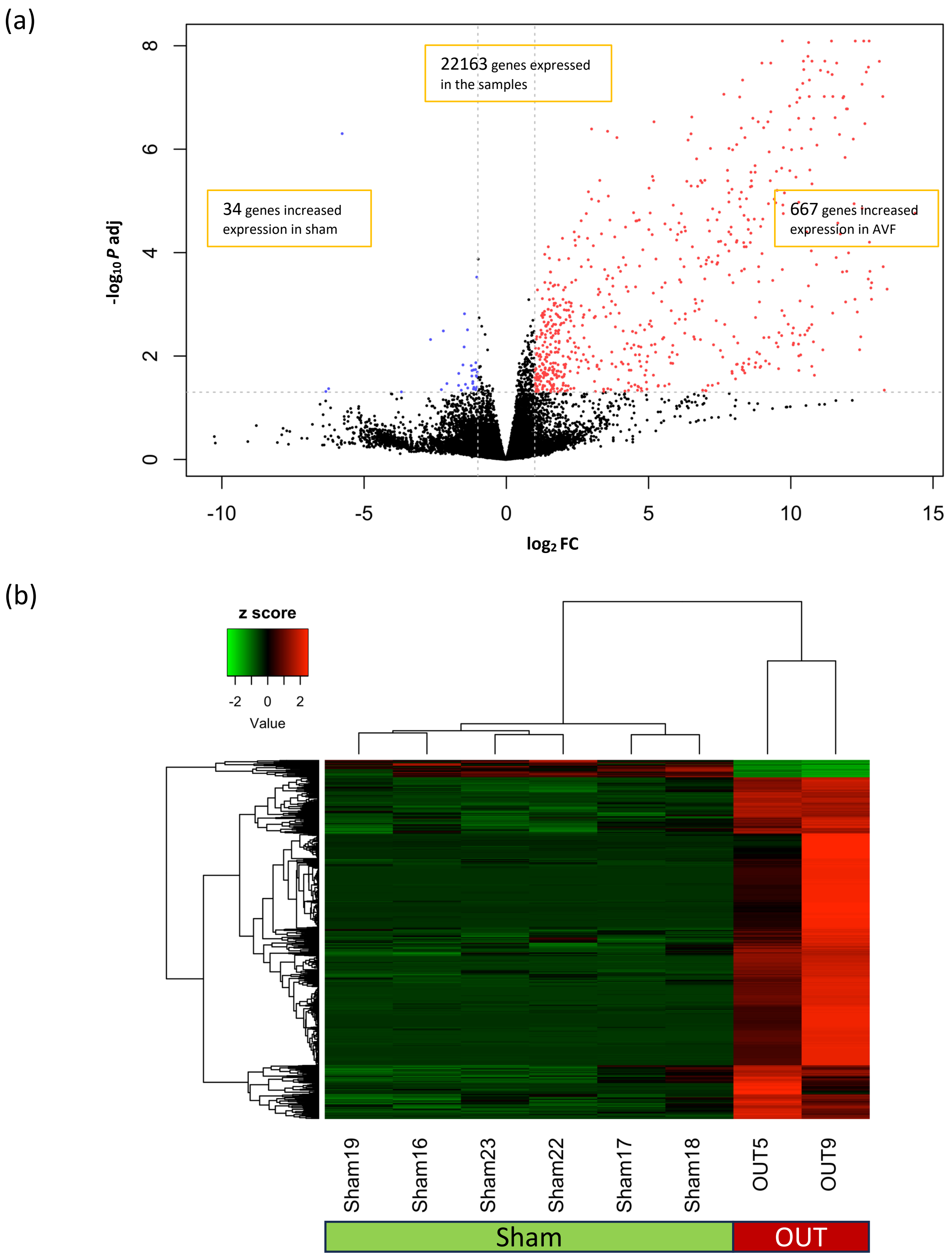

Supplementary Figure S6: Comparison between the sham and OUT groups.

(a) Volcano plot of 22163 genes expressed among the 8 samples. 701 genes were expressed differentially; genes (n=667) with increased expression in the OUT samples are in red dots, and genes (n=34) with increased expression in the nAVF samples are in the blue dots. **(b)** Heatmap plot of the 701 DEG. ***(Continues)***

(c)

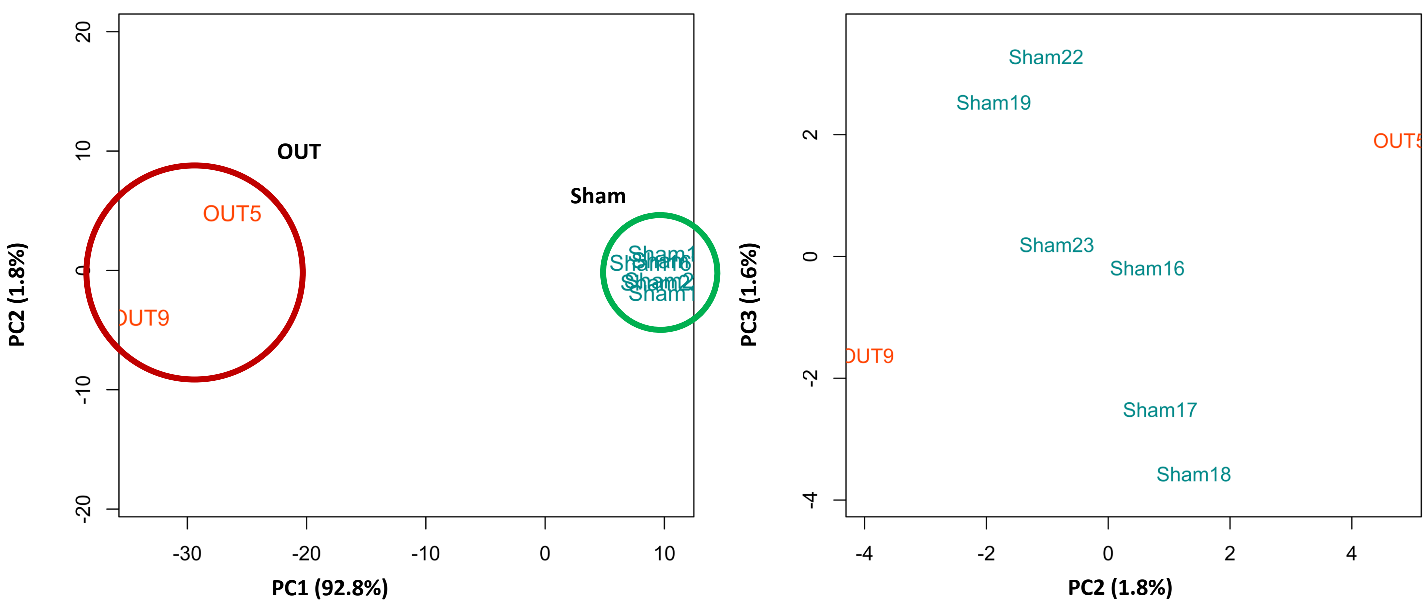

(d)

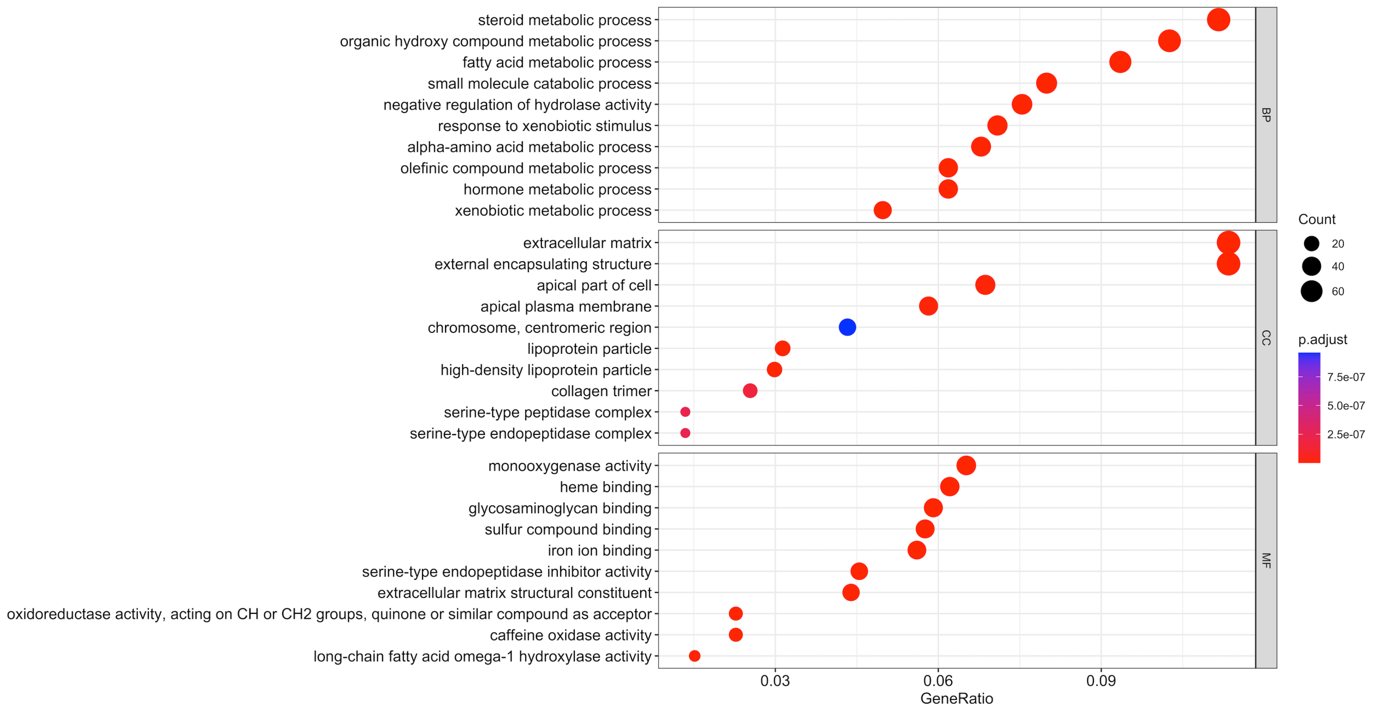

(Continued) Supplementary Figure S6.

(c) Principal component analysis (PCA) plot of the 8 samples (n=6 sham, n=2 OUT) grouping the 701 DEG. The 6 sham samples are circled in green; the 2 OUT samples are circled in red. **(d)** GO enrichment analysis. 302 GO terms (198 BP, 25 CC, and 79 MF) were significantly overrepresented among the 701 DEG. GO terms with the top 10 lowest adjusted P values for each ontology (BP, CC, and MF) are shown. ***(Continues)***

(e)

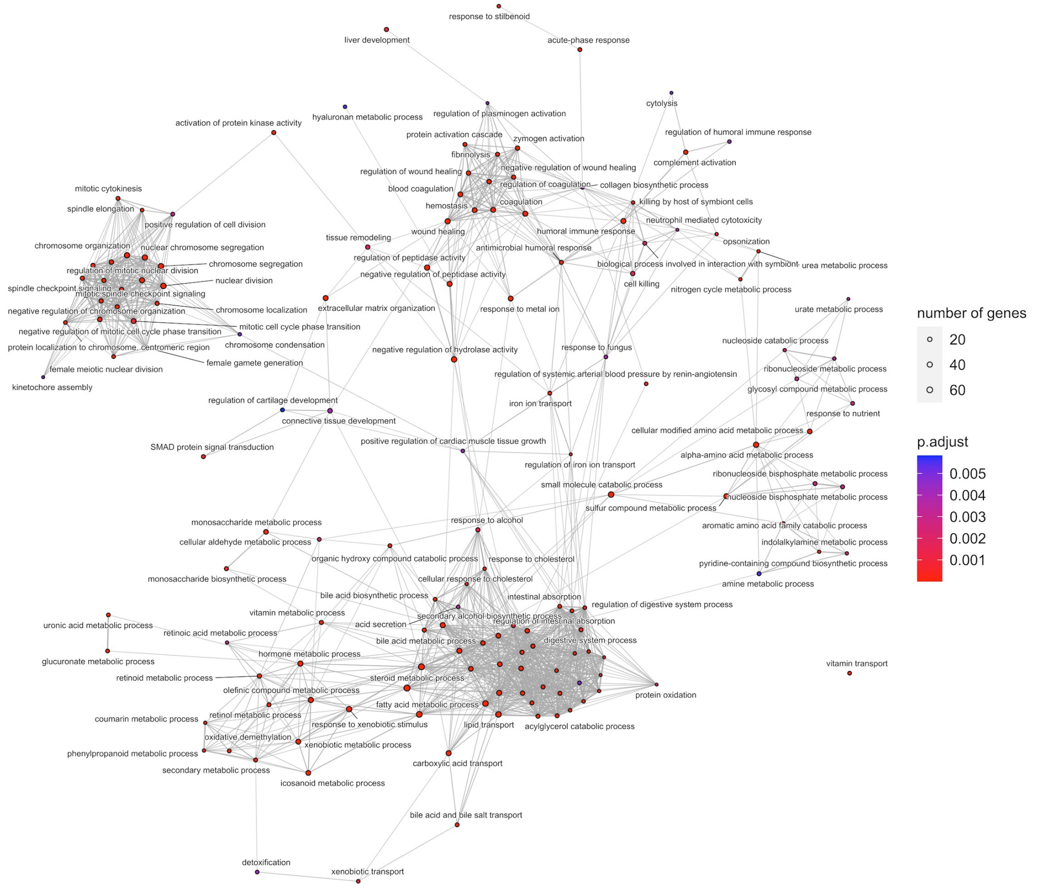

(f)

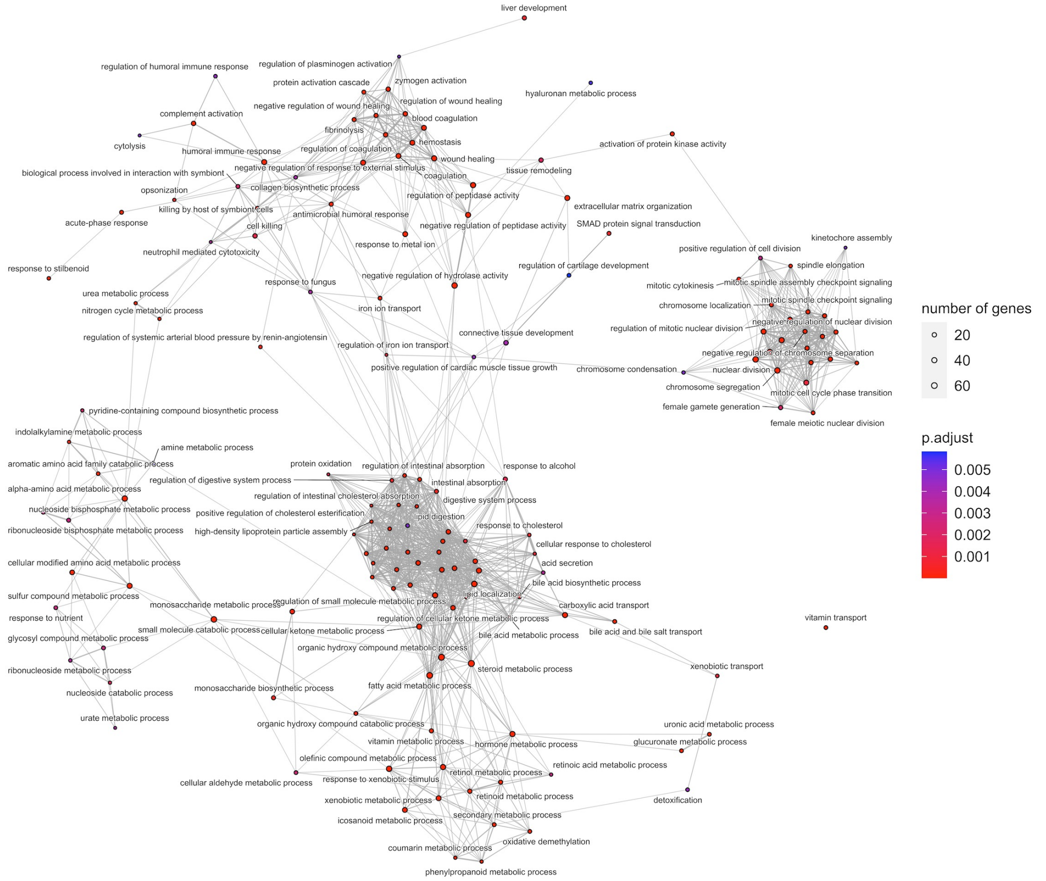

(Continued) Supplementary Figure S6.

(e) Enrichment map plot of the top 150 GO terms. **(f)** Enrichment map plot of the top 150 BP GO terms.

(a)

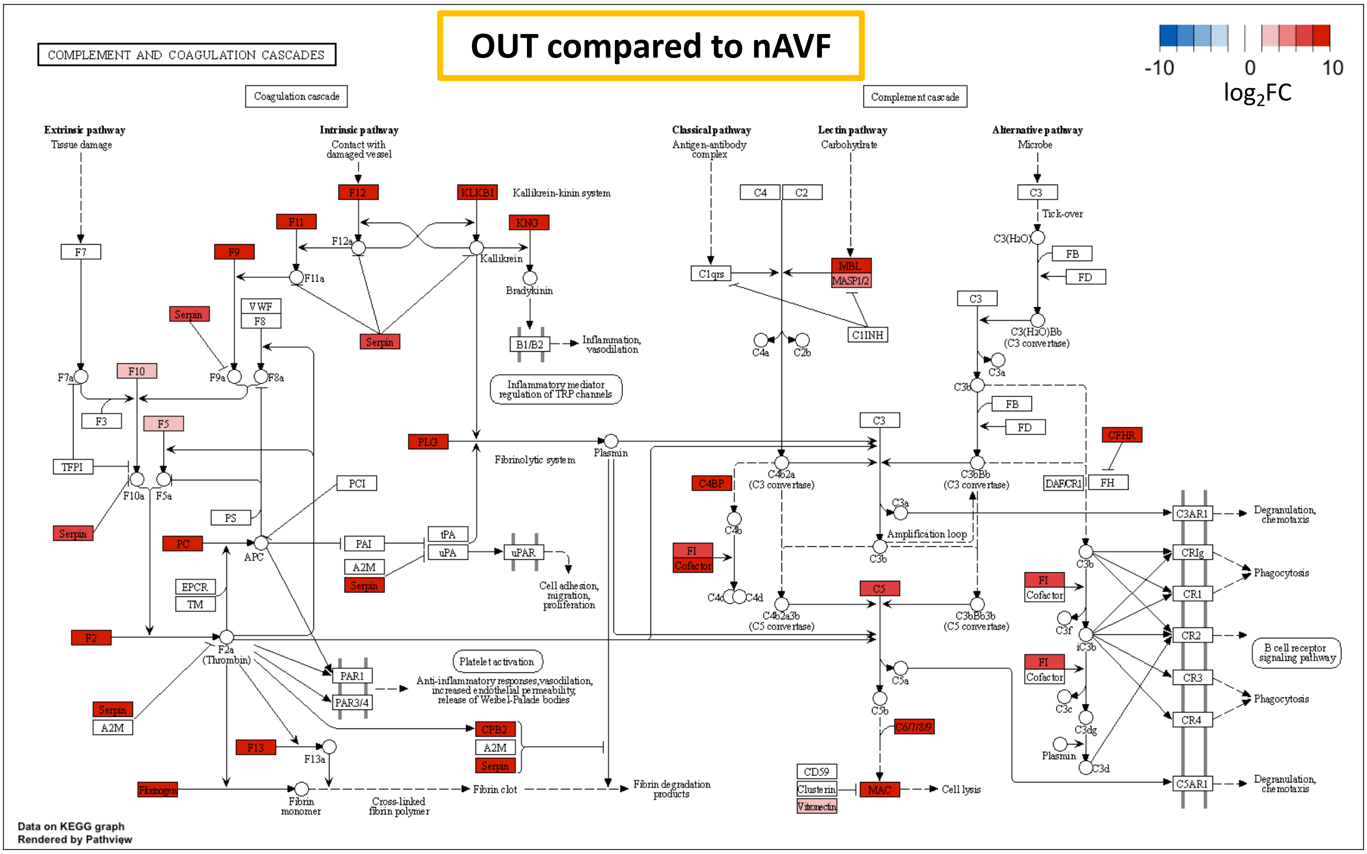

(b)

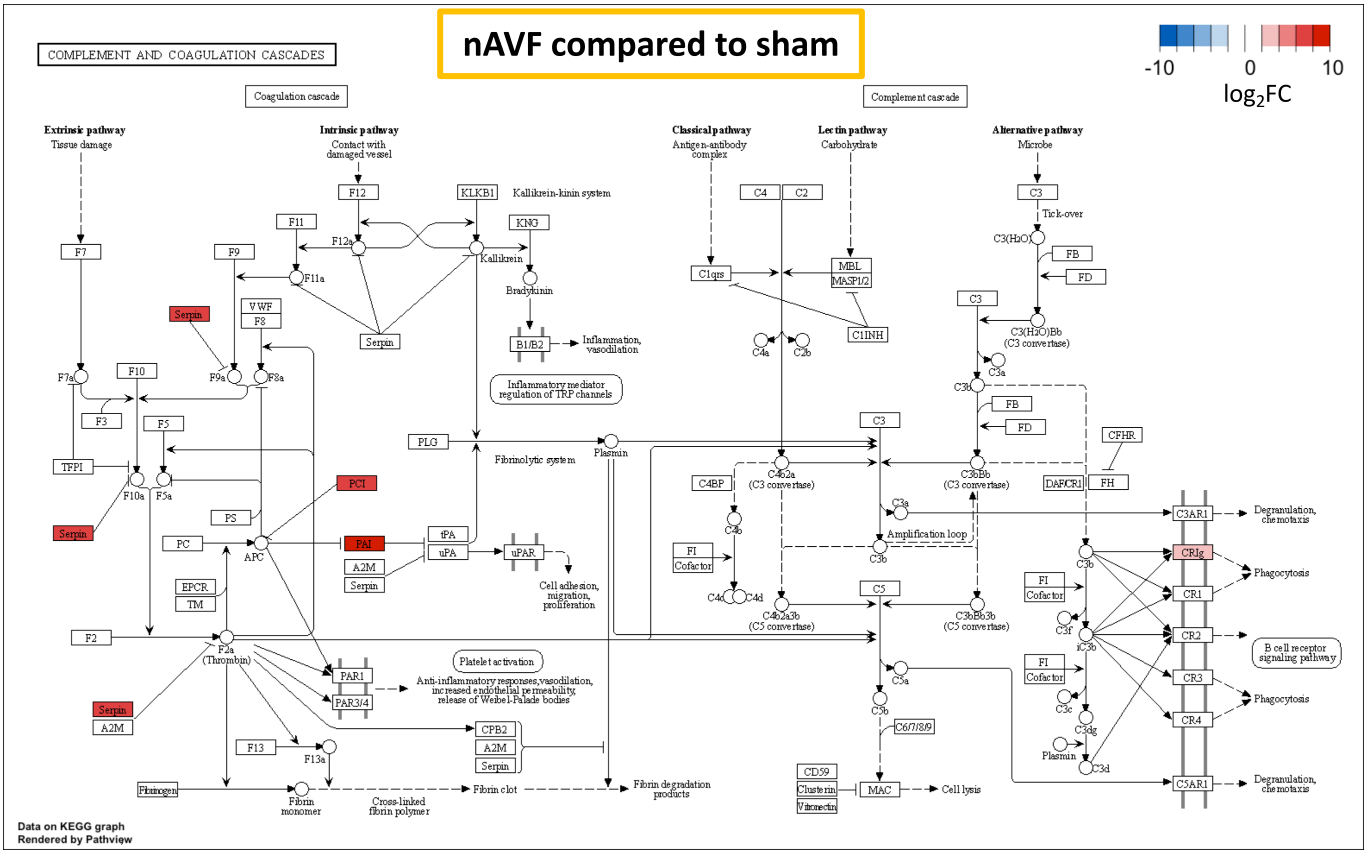

Supplementary Figure S7: Differentially expressed genes (DEG) in the complement and coagulation cascades.

(a) DEG in the OUT group compared to the nAVF group among the complement and coagulation cascades. Red boxes show genes with increased expression in the OUT samples. **(b)** DEG in the nAVF group compared to the sham group among the complement and coagulation cascades. Red boxes show genes with increased expression in the nAVF samples; green boxes show genes with decreased expression in the nAVF samples. ***(Continues)***

(c)

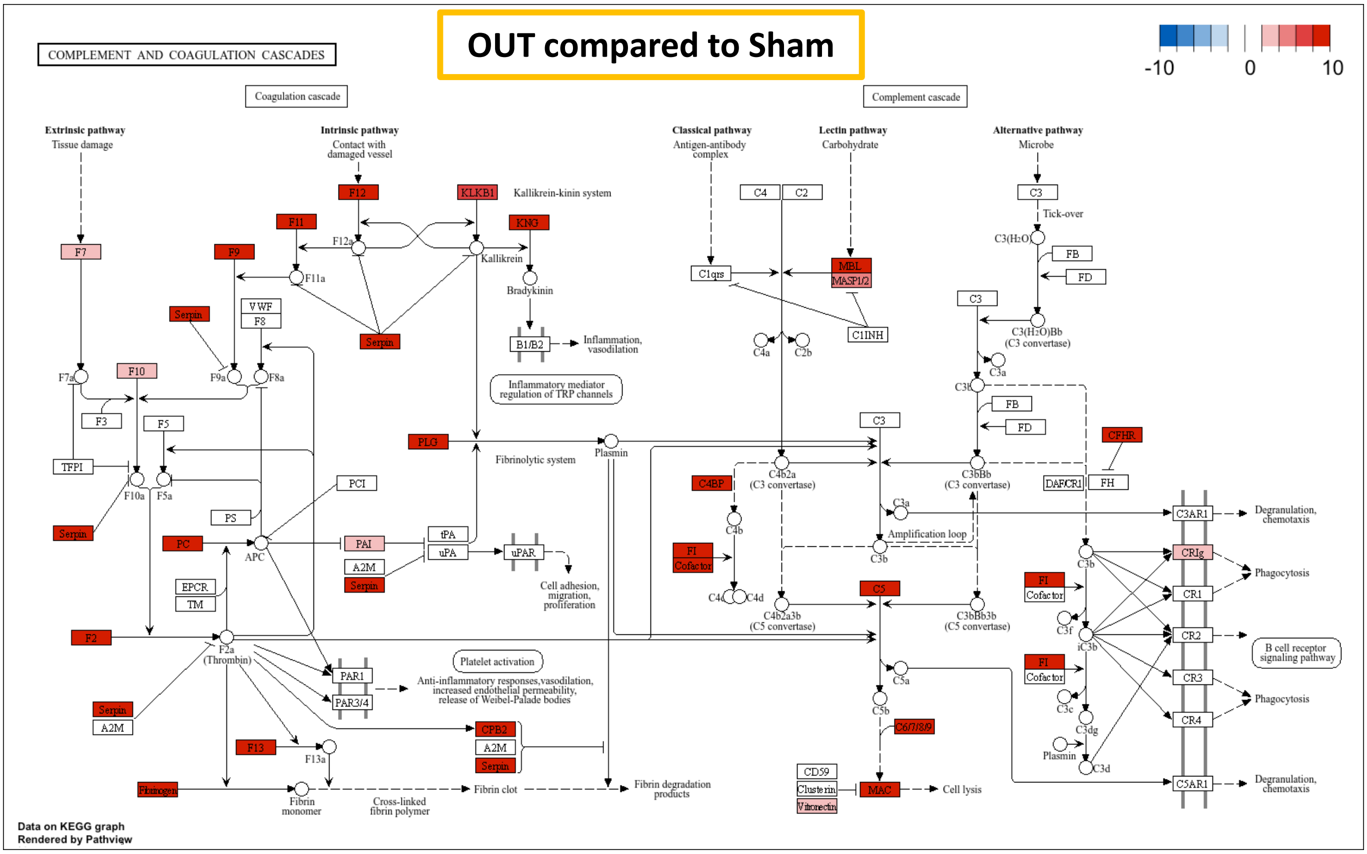

(Continued) Supplementary Figure S7.

(c) DEG in the OUT group compared to the sham group among the complement and coagulation cascades. Red boxes show genes with increased expression in the OUT samples.

(a)

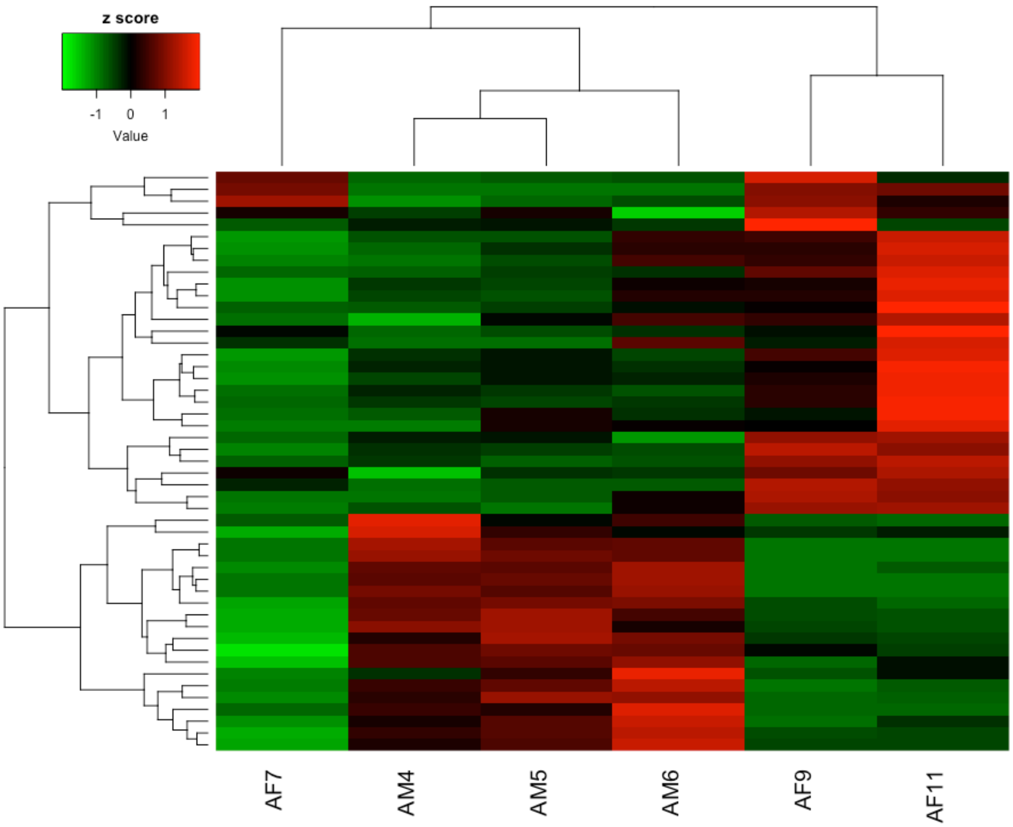

(b)

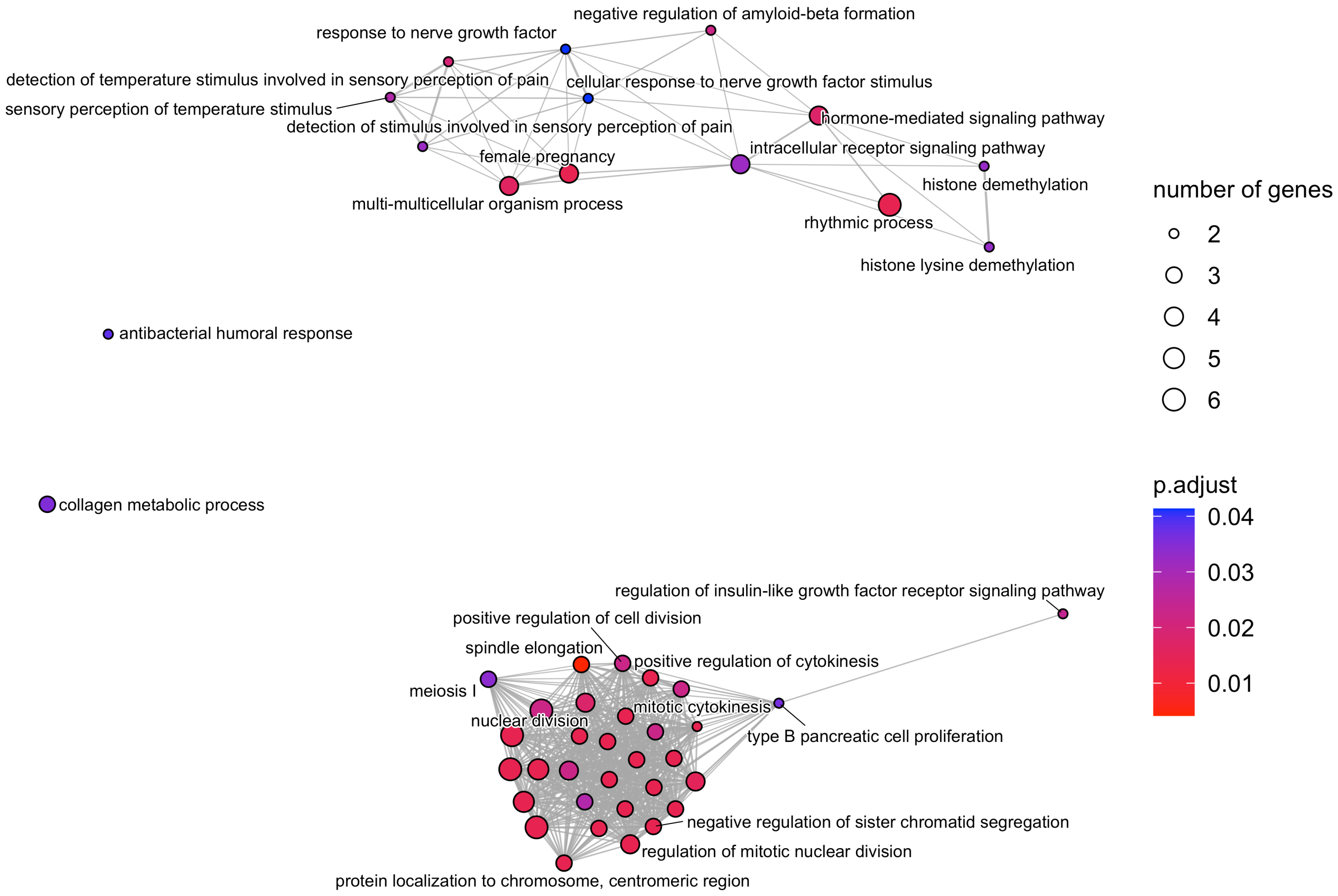

Supplementary Figure S8: Differentially expressed genes (DEG) and enrich analysis between the female and male AVF.
(a) Heatmap plot of the 49 DEG. **(b)** Enrichment map plot of the 47 BP terms. ***(Continues)***

(b)

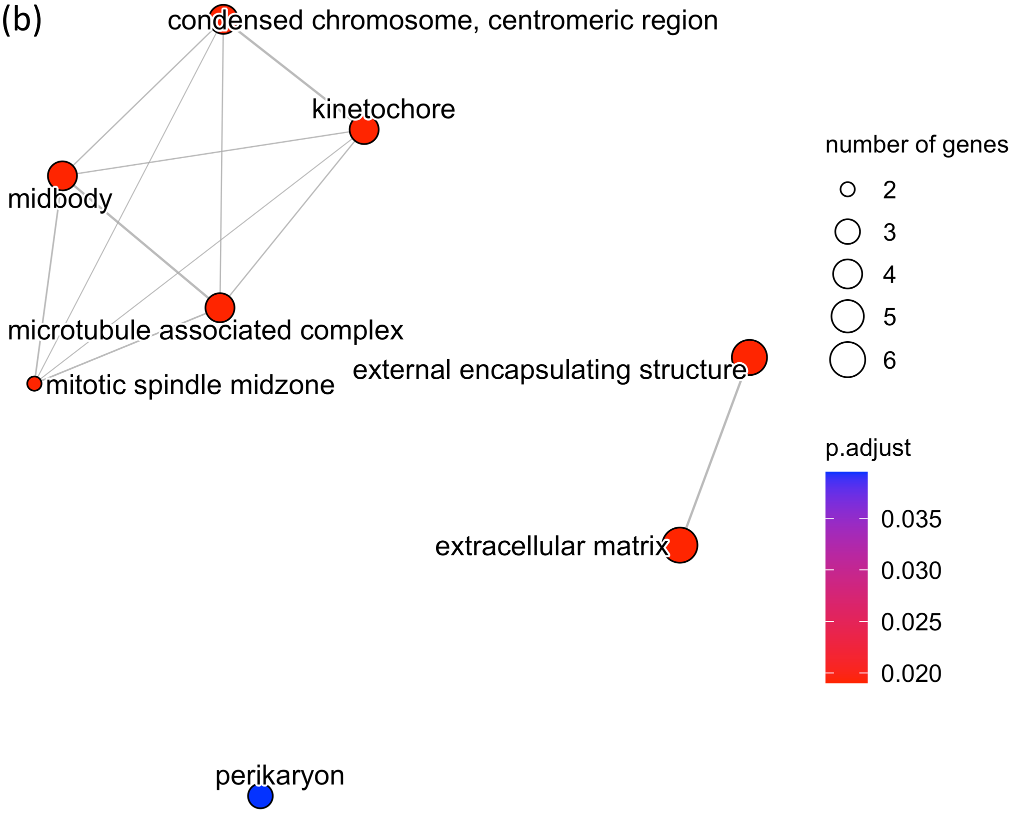

(c)

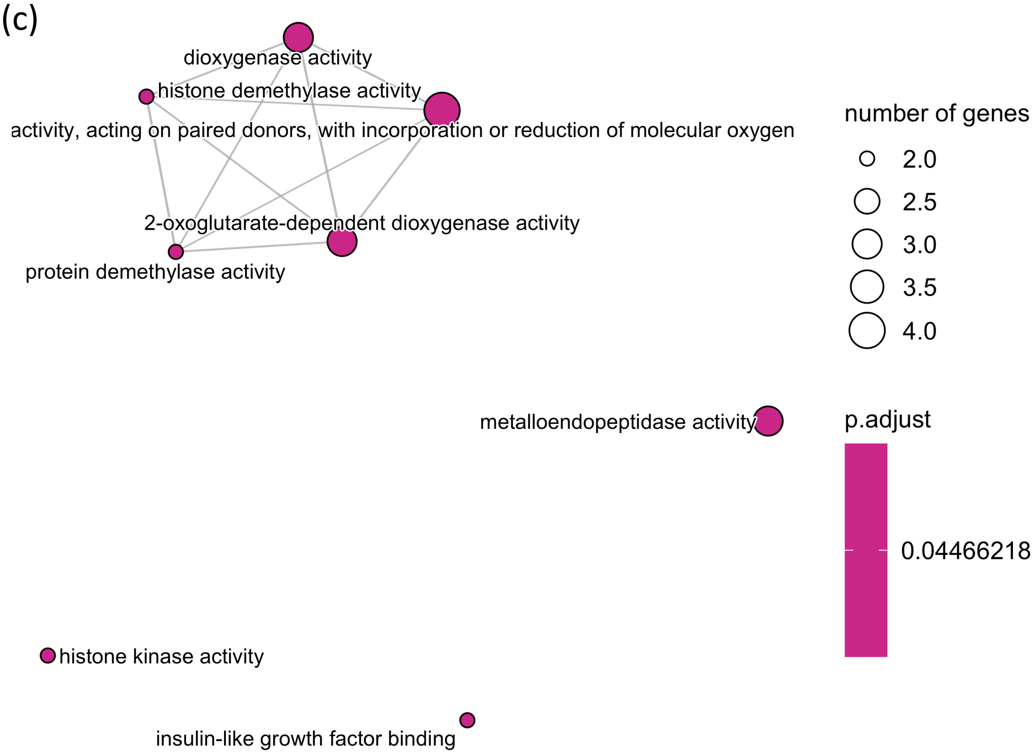

(Continued) Supplementary Figure S8.

(c) Enrichment map plot of the 8 CC terms. **(d)** Enrichment map plot of the 8 MF terms.

Supplementary Table S1. Quality control and mapping result of the RNA sequencing data of the 12 samples.

Sample				Raw data			Quality-controlled data			Mapping	
No.	Group	Sex	RQN	Read Counts	%GC	Sequence Length	Read Counts	%GC	Sequence Length	Uniquely mapped reads	Uniquely mapped %
04	AVF	Male	8.7	28,090,599	45	101	26,592,359	45	36 - 101	21,979,218	82.65
05	AVF	Male	9.0	28,437,777	45	101	27,052,808	45	36 - 101	22,415,830	82.86
06	AVF	Male	9.6	28,469,162	44	101	27,375,604	45	36 - 101	23,403,955	85.49
07	AVF	Female	8.7	27,742,631	49	101	27,029,208	49	36 - 101	22,097,544	81.75
09	AVF	Female	9.0	25,595,425	43	101	24,459,284	44	36 - 101	20,052,835	81.98
11	AVF	Female	10.0	27,397,924	45	101	26,189,494	45	36 - 101	21,937,120	83.76
16	Sham	Male	8.6	30,162,638	43	101	29,003,925	43	36 - 101	23,017,218	79.36
17	Sham	Male	8.9	28,616,513	44	101	27,384,142	44	36 - 101	22,356,447	81.64
18	Sham	Male	9.1	29,328,681	44	101	28,195,833	44	36 - 101	23,679,990	83.98
19	Sham	Female	8.7	27,997,133	43	101	26,653,970	44	36 - 101	21,247,833	79.72
22	Sham	Female	8.3	32,399,920	44	101	31,170,881	44	36 - 101	25,872,045	83.00
23	Sham	Female	9.0	26,136,642	44	101	25,214,238	44	36 - 101	20,649,862	81.90

Supplementary Table S2. MetaCore pathways significantly changed in the microarray analysis of sham and AVF samples

MetaCore Pathway	Gene ratio	(%)	*p* value
Tricarboxylic acid cycle	17/20	85.0	1.26E-14
Propionate metabolism p.2	18/23	78.3	3.06E-14
Butanoate metabolism	17/24	70.8	2.57E-12
Leucine, isoleucine and valine metabolism/ Rodent version	19/30	63.3	2.74E-12
Leucine, isoleucine and valine metabolism.p.2	18/28	64.3	7.25E-12
Propionate metabolism p.1	12/15	80.0	4.77E-10
Regulation of lipid metabolism Insulin regulation of fatty acid metabolism	19/43	44.2	1.10E-08
Mitochondrial long chain fatty acid beta-oxidation	12/18	66.7	1.45E-08
Oxidative phosphorylation	27/83	32.5	2.71E-08
Mitochondrial unsaturated fatty acid beta-oxidation	10/13	76.9	2.90E-08
Cell cycle_Chromosome condensation in prometaphase	12/20	60.0	8.09E-08
Peroxisomal branched chain fatty acid oxidation	12/22	54.5	3.41E-07
Cell cycle_The metaphase checkpoint	15/36	41.7	1.02E-06
Ubiquinone metabolism	17/48	35.4	2.90E-06
Lysine metabolism/ Rodent version	13/30	43.3	3.21E-06
Glycogen metabolism	9/15	60.0	3.74E-06
Cell cycle_Role of APC in cell cycle regulation	13/31	41.9	5.01E-06
Regulation of lipid metabolism_PPAR regulation of lipid metabolism	10/20	50.0	9.52E-06
Tryptophan metabolism/ Rodent version	13/33	39.4	1.15E-05
Mitochondrial ketone bodies biosynthesis and metabolism	7/11	63.6	2.87E-05
Lysine metabolism	11/27	40.7	3.80E-05
Tryptophan metabolism	12/32	37.5	4.49E-05
Regulation of metabolism_Role of Adiponectin in regulation of metabolism	12/37	32.4	2.26E-04
CoA biosynthesis	8/19	42.1	3.46E-04
Cell adhesion_ECM remodeling	14/50	28.0	3.92E-04
Development_Leptin signaling via PI3K-dependent pathway	11/34	32.4	4.23E-04
Pyruvate metabolism/ Rodent version	10/29	34.5	4.34E-04
Mechanism of action of DGAT1 inhibitors in obesity and diabetes mellitus, type II (Variant 1)	6/12	50.0	6.56E-04
Triacylglycerol biosynthesis in obesity and diabetes mellitus, type II	6/12	50.0	6.56E-04
Putative pathways for stimulation of fat cell differentiation by Bisphenol A	9/26	34.6	8.16E-04
Cell cycle_Spindle assembly and chromosome separation	10/32	31.3	1.05E-03
Pyruvate metabolism	8/22	36.4	1.10E-03
Pentose phosphate pathway	6/13	46.2	1.11E-03
Saturated fatty acid biosynthesis	3/3	100.0	1.12E-03
Regulation of lipid metabolism_Insulin regulation of glycogen metabolism	11/39	28.2	1.53E-03
Regulation of lipid metabolism Insulin signaling: generic cascades	11/40	27.5	1.92E-03
Prostaglandin 2 biosynthesis and metabolism	8/24	33.3	2.10E-03
Pentose phosphate pathway/ Rodent version	6/15	40.0	2.70E-03
Cell cycle_Initiation of mitosis	8/25	32.0	2.82E-03

Supplementary Table S3. Metabolic pathways in MetaCore microarray analysis and corresponding RNA-seq genes

Microarray gene name	log$_2$FC	RNA-seq gene name	log$_2$FC	*P* adj
**Tricarboxylic acid cycle**				
IDH3A	-3.3	Idh3a	-1.22	1.42E-03
PYC	-3.2	Pcx	-0.21	8.06E-01
SCS-A	-3.1			
SCS-G	-3.1			
SUCLG1	-3.1	Suclg1	-1.29	1.29E-04
ACON	-3.0	Aco2	-1.33	1.20E-04
SUCB1	-2.9	Dlat	-0.87	1.58E-02
DLDH	-2.7	Dld	-0.65	5.30E-02
ODO1	-2.7	Dhtkd1	0.14	8.72E-01
FUMH	-2.6	Fh1	-0.58	5.48E-02
ODO2	-2.5	Dlst	-0.85	3.57E-03
CISY	-2.4	Cs	-0.75	1.49E-02
Succinate dehydrogenase	-2.2			
IDH3B	-2.2	Idh3b	-1.10	1.59E-04
IDH3	-2.2			
SUCLG2	-2.1	Suclg2	-0.24	5.21E-01
IDH3G	-2.1	Idh3g	-0.81	2.24E-03
**Leucine, isoleucine and valine metabolism/ Rodent version**				
AL1A7	-3.6	Aldh1a7	-0.06	9.54E-01
HADHB	-3.3	Hadhb	-1.30	9.41E-04
ACAA2	-3.0	Acaa2	-1.37	2.22E-05
PCC	-2.7	Acaca	-0.82	1.80E-01
PCCA	-2.7	Pcca	-0.21	7.11E-01
PCCB	-2.7	Pccb	-0.58	2.57E-02
AcetylCoA acyltransferase	-2.7			
ECHS1	-2.7	Echs1	-0.72	2.05E-02
HADHA	-2.7	Hadha	-0.70	3.15E-02
HIBCH	-2.3	Hibch	-0.52	1.52E-01
HCDH	-2.3	Hadh	-0.91	6.40E-03
MCC	-2.3			
MCCC2	-2.3	Mccc2	-0.15	8.02E-01
MUTA	-2.2	Mmut	-0.35	3.18E-01
AUMH	-2.2			
AOX1	-2.1	Aox1	-0.26	5.96E-01
MCCA	-2.1	Mccc1	0.00	9.99E-01
MMSA	-2.0	Aldh6a1	-0.26	6.05E-01
ACAT1	-2.0	Acat1	-0.46	1.18E-01

Microarray gene name	log$_2$FC	RNA-seq gene name	log$_2$FC	*P* adj
**Mitochondrial long chain fatty acid beta-oxidation**				
CPT-1B	-5.9	Cpt1b	-2.35	8.13E-07
ACADVL	-4.0	Acadvl	-1.08	1.68E-03
HADHB	-3.3	Hadhb	-1.30	9.41E-04
ACAA2	-3.1	Acaa2	-1.37	2.22E-05
ACSL5	-2.8	Acsl5	-0.33	2.73E-01
AcetylCoA acyltransferase	-2.7			
ECHS1	-2.7	Echs1	-0.72	2.05E-02
HADHA	-2.7	Hadha	-0.70	3.15E-02
CPT2	-2.6	Cpt2	-0.46	3.09E-01
ACADM	-2.4	Acadm	-0.99	1.14E-02
HCDH	-2.3	Hadh	-0.91	6.40E-03
ACSL1	-2.0	Acsl1	-0.40	5.26E-01
**Oxidative phosphorylation**				
NDUFV1	-2.9	Ndufv1	-0.82	6.91E-03
CYC1	-2.9	Cyc1	-1.02	3.56E-03
UQCRFS1	-2.7	Uqcrfs1	-0.87	1.61E-02
UQCRC1	-2.7	Uqcrc1	-0.70	2.84E-02
NDUFS1	-2.6	Ndufs1	-0.59	4.21E-02
NDUFB8	-2.6	Ndufb8	-1.00	4.83E-04
COX Va	-2.6	Cox5a	-0.95	2.73E-03
UQCR 10	-2.5	Uqcr10	-0.84	9.60E-03
NDUFAB1	-2.4	Ndufab1	-0.90	5.26E-03
NDUFA9	-2.4	Ndufa9	-0.68	1.01E-02
NDUFS6	-2.4	Ndufs6	-0.66	1.94E-02
NDUFA10	-2.3	Ndufa10	-0.92	1.36E-03
NDUFS3	-2.3	Ndufs3	-0.73	1.36E-02
Succinate dehydrogenase	-2.2			
NDUFB6	-2.2	Ndufb6	-0.75	2.47E-02
UQCR11	-2.2	Uqcr11	-0.73	2.42E-02
NDUFS5	-2.2	Ndufs5	-0.65	2.62E-02
NDUFS2	-2.2	Ndufs2	-0.61	4.94E-02
NDUFB9	-2.2	Ndufb9	-0.78	1.24E-02
NDUFS8	-2.1	Ndufs8	-0.68	1.51E-02
ATP5A	-2.1	Atp5a1	-0.62	2.20E-02
NDUFS7	-2.1	Ndufs7	-0.84	1.15E-02
UQCRH	-2.1	Uqcrh	-0.62	1.52E-02
NDUFA4	-2.1	Ndufa4	-0.73	1.31E-02
Cytochrome C	-2.0			
NDUFB5	-2.0	Ndufb5	-0.75	7.33E-03
COX Vb	-2.0	Cox5b	-0.83	2.94E-03

+2.0 **log$_2$FC** -2.0   0.10 **adjusted *P*** 0.005

Supplementary Table S4. Differentially expressed genes comparing the female AVF group vs the male AVF group

Ensembl ID	Gene name	Gene description	Chr.	log_2FC	*P* adj	Order
ENSMUSG086503	Xist	inactive X specific transcripts	X	12.56	1.99E-08	others>AM>SM
ENSMUSG036523	Greb1	gene regulated by estrogen in breast cancer protein	12	3.69	8.75E-03	others>AM>SM
ENSMUSG030854	Ptpn5	protein tyrosine phosphatase, non-receptor type 5	7	2.97	3.94E-02	others>AM
ENSMUSG031870	Pgr	progesterone receptor	9	2.49	7.65E-03	others>AM>SM
ENSMUSG002266	Zim1	zinc finger, imprinted 1	7	2.23	1.27E-02	AF>others
ENSMUSG032783	Troap	trophinin associated protein	15	1.33	4.13E-02	AF>AM>others
ENSMUSG027520	Zdbf2	zinc finger, DBF-type containing 2	1	1.30	3.99E-02	others>AM
ENSMUSG030669	Calca	calcitonin/calcitonin-related polypeptide, alpha	7	1.27	4.69E-02	AF>AM>others
ENSMUSG076934	Iglv1	immunoglobulin lambda variable 1	16	1.19	4.58E-02	AF>AM>others
ENSMUSG027016	Zfp385b	zinc finger protein 385B	2	1.11	1.63E-02	AF>AM>others
ENSMUSG043635	Adamts3	a disintegrin-like and metallopeptidase (reprolysin type) with thrombospondin type 1 motif, 3	5	1.06	7.61E-03	AF>AM>others
ENSMUSG051748	Wfdc21	WAP four-disulfide core domain 21	11	1.05	2.56E-02	AF>AM>others
ENSMUSG038156	Spon1	spondin 1, (f-spondin) extracellular matrix protein	7	1.00	1.26E-03	others>AM>SM
ENSMUSG056749	Nfil3	nuclear factor, interleukin 3, regulated	13	1.00	2.56E-02	AF>others
ENSMUSG109936	Gm45889	predicted gene 45889	7	0.97	3.94E-02	AF>AM>others
ENSMUSG042254	Cilp	cartilage intermediate layer protein, nucleotide pyrophosphohydrolase	9	0.89	2.71E-03	AF>AM>others
ENSMUSG055254	Ntrk2	neurotrophic tyrosine kinase, receptor, type 2	13	0.75	1.16E-02	others>AM
ENSMUSG051048	P4ha3	procollagen-proline, 2-oxoglutarate 4-dioxygenase (proline 4-hydroxylase), alpha polypeptide III	7	0.74	2.01E-02	AF>AM>others
ENSMUSG028370	Pappa	pregnancy-associated plasma protein A	4	0.72	1.62E-03	AF>AM>others
ENSMUSG069910	Spdl1	spindle apparatus coiled-coil protein 1	11	0.70	4.10E-02	AF>AM>others
ENSMUSG091387	Gcnt4	glucosaminyl (N-acetyl) transferase 4, core 2 (beta-1,6-N-acetylglucosaminyltransferase)	13	0.66	8.69E-03	AF>AM>others
ENSMUSG020897	Aurkb	aurora kinase B	11	0.65	1.44E-02	AF>AM>others
ENSMUSG020427	Igfbp3	insulin-like growth factor binding protein 3	11	0.65	6.61E-03	others>AF>AM
ENSMUSG026039	Sgo2a	shugoshin 2A	1	0.65	5.01E-03	AF>AM>others
ENSMUSG050107	Haspin	histone H3 associated protein kinase	11	0.64	2.27E-02	AF>AM>others
ENSMUSG046178	Nxph1	neurexophilin 1	6	0.63	4.07E-02	AF>AM>others
ENSMUSG034311	Kif4	kinesin family member 4	X	0.60	1.61E-03	AF>AM>others
ENSMUSG024795	Kif20b	kinesin family member 20B	19	0.60	5.87E-03	AF>AM>others
ENSMUSG017716	Birc5	baculoviral IAP repeat-containing 5	11	0.59	2.72E-03	AF>AM>others
ENSMUSG017969	Ptgis	prostaglandin I2 (prostacyclin) synthase	2	-0.64	1.99E-02	AM>AF>others
ENSMUSG022610	Mapk12	mitogen-activated protein kinase 12	15	-0.75	7.28E-03	others>AM>AF
ENSMUSG031075	Ano1	anoctamin 1, calcium activated chloride channel	7	-0.81	3.99E-02	others>AF
ENSMUSG017737	Mmp9	matrix metallopeptidase 9	2	-0.83	4.35E-02	others>AM>AF
ENSMUSG022389	Tef	thyrotroph embryonic factor	15	-0.87	2.61E-02	others>AF
ENSMUSG027656	Ccn5	cellular communication network factor 5	2	-0.90	1.87E-02	AM>AF>others
ENSMUSG118633	Gm21104	predicted gene, 21104	4	-0.90	1.69E-02	AM>others
ENSMUSG021775	Nr1d2	nuclear receptor subfamily 1, group D, member 2	14	-0.96	1.09E-02	others>AF
ENSMUSG039457	Ppl	periplakin	16	-1.05	1.90E-02	others>AF
ENSMUSG051504	Siglech	sialic acid binding Ig-like lectin H	7	-1.14	3.41E-02	others>AF
ENSMUSG071317	Bves	blood vessel epicardial substance	10	-1.18	3.11E-02	others>AM>AF
ENSMUSG079465	Col4a3	collagen, type IV, alpha 3	1	-1.48	1.40E-02	AM>SM>others
ENSMUSG059824	Dbp	D site albumin promoter binding protein	7	-1.84	1.65E-02	others>AF
ENSMUSG067001	Serpinb7	serine (or cysteine) peptidase inhibitor, clade B, member 7	1	-3.08	2.71E-03	others>AF>SF
ENSMUSG109237	9130214F15Rik	RIKEN cDNA 9130214F15 gene	8	-3.64	2.92E-02	others>AM>AF
ENSMUSG099876	Gm29650	predicted gene 29650	Y	-6.04	2.21E-02	SM>AM>others
ENSMUSG056673	Kdm5d	lysine (K)-specific demethylase 5D	Y	-9.75	4.36E-06	others>AF>SF
ENSMUSG068457	Uty	ubiquitously transcribed tetratricopeptide repeat containing, Y-linked	Y	-12.21	1.33E-05	AM>SM>others
ENSMUSG069049	Eif2s3y	eukaryotic translation initiation factor 2, subunit 3, structural gene Y-linked	Y	-12.83	4.36E-06	others>AF=SF
ENSMUSG069045	Ddx3y	DEAD box helicase 3, Y-linked	Y	-14.45	1.14E-06	others>SF>AF

Supplementary Code S1. Quality control, mapping and counting.

```
module load PRINSEQ/0.20.4-foss-2018b-Perl-5.28.0
module load Trimmomatic/0.39-Java-11
module load STAR/2.7.9a-GCCcore-10.2.0
module load RSEM/1.3.3-foss-2020b

#poly A/T removal
for i in ${SampleNo[@]}
do
        prinseq-lite.pl
         -verbose -fastq ${mainpath}/FASTQs/${i}R1.fastq -fastq2 ${mainpath}/FASTQs/${i}R2.fastq \
         -trim_tail_right 6 -trim_tail_left 6 -out_format 3 \
         -out_good ${mainpath}/TrimmedFQ/${i}good -out_bad ${mainpath}/TrimmedFQ/${i}bad
done

#Adapters and low-quality reads removal
for i in ${SampleNo[@]}
do
        java -jar $EBROOTTRIMMOMATIC/trimmomatic-0.39.jar \
         PE -threads 64 -phred33 \
         ${mainpath}/TrimmedFQ/${i}good_1.fastq ${mainpath}/TrimmedFQ/${i}good_2.fastq \
         ${mainpath}/TrimmedFQ/${i}R1clean.fastq.gz ${mainpath}/TrimmedFQ/${i}R1unpaired.fastq.gz \
         ${mainpath}/TrimmedFQ/${i}R2clean.fastq.gz ${mainpath}/TrimmedFQ/${i}R2unpaired.fastq.gz \
         ILLUMINACLIP:${mainpath}/COMMANDS/AdaptorSeq.fa:2:30:10 \
         LEADING:30 TRAILING:30 SLIDINGWINDOW:4:15 MINLEN:36
done


#Index creation
STAR --runMode genomeGenerate \
         --genomeDir ${mainpath}/Index --runThreadN 64 --limitGenomeGenerateRAM 99000000000 --sjdbOverhang 100 \
         --genomeFastaFiles ${mainpath}/GRCm39.fa --sjdbGTFfile ${mainpath}/GRCm39.gtf

#Mapping
for i in ${SampleNo[@]}
do
        STAR --outSAMtype BAM SortedByCoordinate --quantMode TranscriptomeSAM --runThreadN 64 --genomeLoad NoSharedMemory --outSAMattributes All \
         --readFilesCommand gunzip -c --genomeDir ${mainpath}/Index
         --readFilesIn ${mainpath}/TrimmedFQ/${i}R1clean.fastq.gz ${mainpath}/TrimmedFQ/${i}R2clean.fastq.gz \
         --outFileNamePrefix ${mainpath}/BAMs/${i}.
done

#Reference Creation
rsem-prepare-reference --gtf ${mainpath}/COMMANDS/GRCm39.gtf -p 64 ${mainpath}/COMMANDS/GRCm39.fa ${mainpath}/Index/GRCm39

#Counting gene expression amounts
for i in ${SampleNo[@]}
do
        rsem-calculate-expression --paired-end --alignments --estimate-rspd --append-names --strandedness reverse -p 64
         ${mainpath}/BAMs/${i}.Aligned.toTranscriptome.out.bam ${mainpath}/Index/GRCm39   ${mainpath}/Counts/${i}
done
```

Supplementary Code S2. DEG analysis and GO enrichment analysis comparing the sham and AVF groups

```
library(edgeR)
library(clusterProfiler)
library(org.Mm.eg.db)

#load count data
count <- read.table("CountsResult.tsv", sep="\t", header=T, row.names=1, quote="")
count <- as.matrix(count)

#DEG analysis
group <- factor(c("AVF", "AVF", "AVF", "AVF", "AVF", "AVF", "Sham", "Sham", "Sham", "Sham", "Sham", "Sham"), levels=c("Sham", "AVF"))
d <- DGEList(counts=count, group=group)
cpm <- cpm(d)
keep <- rowSums(cpm>1)>=2
d <- d[keep,]
d <- calcNormFactors(d)
d <- estimateCommonDisp(d)
d <- estimateTagwiseDisp(d)
exact <- exactTest(d)
table <- as.data.frame(topTags(exact, n=nrow(data), sort.by="none"))
table$gene_id <- row.names(table)
write.table(table, file="DEG.tsv", row.names=T, col.names=T, sep="\t", quote=F)

#GO enrichment analysis
table <- read.table("DEG.tsv", sep="\t", header=T, row.names=1, quote="")
all.genes <- rownames(table)
is.degs <- all.genes[table$FDR < 0.05 & abs(table$logFC) >= 1]
all.genes.entrez <- bitr(all.genes, fromType = "ENSEMBL", toType = "ENTREZID", OrgDb = "org.Mm.eg.db")
is.degs.entrez <- bitr(is.degs, fromType = "ENSEMBL", toType = "ENTREZID", OrgDb = "org.Mm.eg.db")
ego <- enrichGO(gene = is.degs.entrez[,2], universe = all.genes.entrez[,2], OrgDb = org.Mm.eg.db, ont = "ALL",
                pAdjustMethod = "BH", pvalueCutoff = 0.05, qvalueCutoff = 1, readable = TRUE)
ego.result <- simplify(ego)
write.table(ego.result, ”GO.tsv", sep="\t", row.names=F, col.names = T, quote = F)
```

Supplementary Code S3. Subgroup analyses between the nAVF and OUT groups.

```
library(TCC)
library(baySeq)
library(edgeR)
library(clusterProfiler)
library(org.Mm.eg.db)

#subgroup analysis Sham-nAVF-OUT
param_OUT <- 2
param_nAVF <- 4
param_Sham <- 6
param_order <- c("nonDEG", "DEG_OUT", "DEG_nAVF", "DEG_Sham", "DEGall")
param_samplesize <- 100000

data <- read.table(CountsResult.tsv, header=T, row.names=1, sep="\t", quote="")
data <- data[!(rowSums(data)<=12),]
data <- round(data)
data.cl <- c(rep(1, param_OUT), rep(2, param_nAVF), rep(3, param_Sham))
tcc <- new("TCC", data, data.cl)
tcc <- calcNormFactors(tcc, norm.method="tmm", test.method="edger", iteration=3, FDR=0.1, floorPDEG=0.05)

ba <- new("countData", data=as.matrix(data), replicates=data.cl)
nonDEG <- factor(c(rep("non", param_OUT), rep("non", param_nAVF), rep("non", param_Sham)))
DEG_OUT <- factor(c(rep("OUT", param_OUT), rep("other", param_nAVF), rep("other", param_Sham)))
DEG_nAVF <- factor(c(rep("other", param_OUT), rep("nAVF", param_nAVF), rep("other", param_Sham)))
DEG_Sham <- factor(c(rep("other", param_OUT), rep("other", param_nAVF), rep("Sham", param_Sham)))
DEGall <- factor(c(rep("OUT", param_OUT), rep("nAVF", param_nAVF), rep("Sham", param_Sham)))
groups(ba) <- list(nonDEG = nonDEG, DEG_OUT = DEG_OUT, DEG_nAVF = DEG_nAVF, DEG_Sham = DEG_Sham, DEGall =DEGall)

tcc <- estimateDE(tcc, test.method="edger", FDR=0.05)
result <- getResult(tcc, sort=FALSE)
sum(tcc$stat$q.value < 0.05)
libsizes(ba) <- colSums(data)*tcc$norm.factors
ba <- getPriors.NB(ba, samplesize=param_samplesize, estimation="QL", cl=NULL)
ba <- getLikelihoods(ba, pET="BIC", nullData=FALSE, cl=NULL)

out <- list()
out$PP <- exp(ba@posteriors)
out$MAP <- param_order[max.col(out$PP)]
ranking <- tcc$stat$rank
orderings <- NULL
for(i in 1:length(out$MAP)){
  orderings <- append(orderings, as.character(ba@orderings[i, max.col(out$PP)[i]]))
}

tmp <- cbind(rownames(data), data, out$PP, out$MAP, orderings, ranking, result$q.value)
write.table(tmp, DEG-ANOVA.tsv, sep="\t", append=F, quote=F, row.names=F)

#Fold Change calculation
count <- read.table("CountsResult-nAVF-OUT.tsv", sep="\t", header=TRUE, row.names=1, quote="")
count <- as.matrix(count)
group <- factor(c("OUT", "OUT", "nAVF", "nAVF", "nAVF", "nAVF"), levels=c("nAVF", "OUT"))

d <- DGEList(counts=count, group=group)
d <- calcNormFactors(d)
d <- estimateCommonDisp(d)
d <- estimateTagwiseDisp(d)
exact <- exactTest(d)
table <- as.data.frame(topTags(exact, n=nrow(data), sort.by = "none"))
write.table(table, file="FC-ANOVA.tsv", sep="\t", row.names = TRUE, col.names = TRUE, quote=F)

#GO ANALYSIS
table <- read.table("DEG-FC-ANOVA.tsv", sep="\t", header=T, row.names=1, quote="")
all.genes <- rownames(table)
is.degs <- all.genes[table$qvalue < 0.05 & abs(table$logFC) >= 1]

all.genes.entrez <- bitr(all.genes, fromType = "ENSEMBL", toType = "ENTREZID", OrgDb = "org.Mm.eg.db")
is.degs.entrez <- bitr(is.degs, fromType = "ENSEMBL", toType = "ENTREZID", OrgDb = "org.Mm.eg.db")

ego <- enrichGO(gene = is.degs.entrez[,2], universe = all.genes.entrez[,2], OrgDb = org.Mm.eg.db, ont = "ALL",
                            pAdjustMethod = "BH", pvalueCutoff = 0.05, qvalueCutoff = 1, readable = TRUE)
ego.result <- simplify(ego)
write.table(ego.result, "GO-nAVF-OUT.tsv", sep="\t", row.names=F, col.names = T, quote = F)

#KEGG pathway analysis
kegg.result <- enrichKEGG(gene=as.character(is.degs.entrez[,2]), universe=as.character(all.genes.entrez[,2]), keyType = "ncbi-geneid", organism="mmu",
             pvalueCutoff =  0.05, qvalueCutoff = 1, pAdjustMethod = "BH", use_internal_data = F)
write.table(kegg.result, "KEGG-nAVF-OUT.tsv", sep="\t", row.names=F, col.names = T, quote = F)
```

Supplementary Code S4. Subgroup analyses between the sham and nAVF groups.

```
library(TCC)
library(baySeq)
library(edgeR)
library(clusterProfiler)
library(org.Mm.eg.db)

#subgroup analysis Sham-nAVF-OUT
param_OUT <- 2
param_nAVF <- 4
param_Sham <- 6
param_order <- c("nonDEG", "DEG_OUT", "DEG_nAVF", "DEG_Sham", "DEGall")
param_samplesize <- 100000

data <- read.table(../CountsResult.tsv, header=T, row.names=1, sep="\t", quote="")
data <- data[!(rowSums(data)<=12),]
data <- round(data)
data.cl <- c(rep(1, param_OUT), rep(2, param_nAVF), rep(3, param_Sham))
tcc <- new("TCC", data, data.cl)
tcc <- calcNormFactors(tcc, norm.method="tmm", test.method="edger", iteration=3, FDR=0.1, floorPDEG=0.05)

ba <- new("countData", data=as.matrix(data), replicates=data.cl)
nonDEG <- factor(c(rep("non", param_OUT), rep("non", param_nAVF), rep("non", param_Sham)))
DEG_OUT <- factor(c(rep("OUT", param_OUT), rep("other", param_nAVF), rep("other", param_Sham)))
DEG_nAVF <- factor(c(rep("other", param_OUT), rep("nAVF", param_nAVF), rep("other", param_Sham)))
DEG_Sham <- factor(c(rep("other", param_OUT), rep("other", param_nAVF), rep("Sham", param_Sham)))
DEGall <- factor(c(rep("OUT", param_OUT), rep("nAVF", param_nAVF), rep("Sham", param_Sham)))
groups(ba) <- list(nonDEG = nonDEG, DEG_OUT = DEG_OUT, DEG_nAVF = DEG_nAVF, DEG_Sham = DEG_Sham, DEGall =DEGall)

tcc <- estimateDE(tcc, test.method="edger", FDR=0.05)
result <- getResult(tcc, sort=FALSE)
sum(tcc$stat$q.value < 0.05)
libsizes(ba) <- colSums(data)*tcc$norm.factors
ba <- getPriors.NB(ba, samplesize=param_samplesize, estimation="QL", cl=NULL)
ba <- getLikelihoods(ba, pET="BIC", nullData=FALSE, cl=NULL)

out <- list()
out$PP <- exp(ba@posteriors)
out$MAP <- param_order[max.col(out$PP)]
ranking <- tcc$stat$rank
orderings <- NULL
for(i in 1:length(out$MAP)){
  orderings <- append(orderings, as.character(ba@orderings[i, max.col(out$PP)[i]]))
}

tmp <- cbind(rownames(data), data, out$PP, out$MAP, orderings, ranking, result$q.value)
write.table(tmp, DEG-ANOVA.tsv, sep="\t", append=F, quote=F, row.names=F)

#Fold Change calculation
count <- read.table("CountsResult-Sham-nAVF.tsv", sep="\t", header=TRUE, row.names=1, quote="")
count <- as.matrix(count)
group <- factor(c("nAVF", "nAVF", "nAVF", "nAVF", "Sham", "Sham", "Sham", "Sham", "Sham", "Sham"), levels=c("Sham", "nAVF"))

d <- DGEList(counts=count, group=group)
d <- calcNormFactors(d)
d <- estimateCommonDisp(d)
d <- estimateTagwiseDisp(d)
exact <- exactTest(d)
table <- as.data.frame(topTags(exact, n=nrow(data), sort.by = "none"))
write.table(table, file="FC-ANOVA.tsv", sep="\t", row.names = TRUE, col.names = TRUE, quote=F)

#GO ANALYSIS
table <- read.table("DEG-FC-Sham-nAVF.tsv", sep="\t", header=T, row.names=1, quote="")
all.genes <- rownames(table)
is.degs <- all.genes[table$qvalue < 0.05 & abs(table$logFC) >= 1]

all.genes.entrez <- bitr(all.genes, fromType = "ENSEMBL", toType = "ENTREZID", OrgDb = "org.Mm.eg.db")
is.degs.entrez <- bitr(is.degs, fromType = "ENSEMBL", toType = "ENTREZID", OrgDb = "org.Mm.eg.db")

ego <- enrichGO(gene = is.degs.entrez[,2], universe = all.genes.entrez[,2], OrgDb = org.Mm.eg.db, ont = "ALL",
                                   pAdjustMethod = "BH", pvalueCutoff = 0.05, qvalueCutoff = 1, readable = TRUE)
ego.result <- simplify(ego)
write.table(ego.result, "GO-Sham-nAVF.tsv", sep="\t", row.names=F, col.names = T, quote = F)

#KEGG pathway analysis
kegg.result <- enrichKEGG(gene=as.character(is.degs.entrez[,2]), universe=as.character(all.genes.entrez[,2]), keyType = "ncbi-geneid", organism="mmu",
                    pvalueCutoff =  0.05, qvalueCutoff = 1, pAdjustMethod = "BH", use_internal_data = F)
write.table(kegg.result, "KEGG-Sham-nAVF.tsv", sep="\t", row.names=F, col.names = T, quote = F)
```

Supplementary Code S5. Subgroup analyses of the sex difference.

```
library(TCC)
library(baySeq)
library(edgeR)
library(clusterProfiler)
library(org.Mm.eg.db)

#subgroup analysis for Sex differences
param_AM <- 3
param_AF <- 3
param_SM <- 3
param_SF <- 3
param_order <- c("nonDEG", "DEG_AM", "DEG_AF", "DEG_SM", "DEG_SF", "DEG_AMAF", "DEG_AMSM", "DEG_AMSF", "DEG_AFSM", "DEG_AFSF", "DEG_SMSF", "DEGall")
param_samplesize <- 100000

data <- read.table(../CountsResult.tsv, header=T, row.names=1, sep="\t", quote="")
data <- data[!(rowSums(data)<=12),]
data <- round(data)
data.cl <- c(rep(1, param_AM), rep(2, param_AF), rep(3, param_SM), rep(4, param_SF))
tcc <- new("TCC", data, data.cl)
tcc <- calcNormFactors(tcc, norm.method="tmm", test.method="edger", iteration=3, FDR=0.1, floorPDEG=0.05)

ba <- new("countData", data=as.matrix(data), replicates=data.cl)
nonDEG <- factor(c(rep("non",param_AM), rep("non",param_AF), rep("non",param_SM), rep("non",param_SF)))
DEG_AM <- factor(c(rep("AM",param_AM), rep("other",param_AF), rep("other",param_SM), rep("other",param_SF)))
DEG_AF <- factor(c(rep("other",param_AM), rep("AF",param_AF), rep("other",param_SM), rep("other",param_SF)))
DEG_SM <- factor(c(rep("other",param_AM), rep("other",param_AF), rep("SM",param_SM), rep("other",param_SF)))
DEG_SF <- factor(c(rep("other",param_AM), rep("other",param_AF), rep("other",param_SM), rep("SF",param_SF)))
DEG_AMAF <- factor(c(rep("AM",param_AM), rep("AF",param_AF), rep("other",param_SM), rep("other",param_SF)))
DEG_AMSM <- factor(c(rep("AM",param_AM), rep("other",param_AF), rep("SM",param_SM), rep("other",param_SF)))
DEG_AMSF <- factor(c(rep("AM",param_AM), rep("other",param_AF), rep("other",param_SM), rep("SF",param_SF)))
DEG_AFSM <- factor(c(rep("other",param_AM), rep("AF",param_AF), rep("SM",param_SM), rep("other",param_SF)))
DEG_AFSF <- factor(c(rep("other",param_AM), rep("AF",param_AF), rep("other",param_SM), rep("SF",param_SF)))
DEG_SMSF <- factor(c(rep("other",param_AM), rep("other",param_AF), rep("SM",param_SM), rep("SF",param_SF)))
DEGall <- factor(c(rep("AM",param_AM), rep("AF",param_AF), rep("SM",param_SM), rep("SF",param_SF)))
groups(ba) <- list(nonDEG = nonDEG, DEG_AM = DEG_AM, DEG_AF = DEG_AF, DEG_SM = DEG_SM, DEG_SF = DEG_SF, DEG_AMAF = DEG_AMAF,
                   DEG_AMSM = DEG_AMSM, DEG_AMSF = DEG_AMSF, DEG_AFSM = DEG_AFSM, DEG_AFSF = DEG_AFSF, DEG_SMSF = DEG_SMSF, DEGall = DEGall)

tcc <- estimateDE(tcc, test.method="edger", FDR=0.05)
result <- getResult(tcc, sort=FALSE)
sum(tcc$stat$q.value < 0.05)
libsizes(ba) <- colSums(data)*tcc$norm.factors
ba <- getPriors.NB(ba, samplesize=param_samplesize, estimation="QL", cl=NULL)
ba <- getLikelihoods(ba, pET="BIC", nullData=FALSE, cl=NULL)

out <- list()
out$PP <- exp(ba@posteriors)
out$MAP <- param_order[max.col(out$PP)]
ranking <- tcc$stat$rank
orderings <- NULL
for(i in 1:length(out$MAP)){
  orderings <- append(orderings, as.character(ba@orderings[i, max.col(out$PP)[i]]))
}

tmp <- cbind(rownames(data), data, out$PP, out$MAP, orderings, ranking, result$q.value)
write.table(tmp, DEG-SexDifference.tsv, sep="\t", append=F, quote=F, row.names=F)

#Fold Change calculation
count <- read.table("CountsResult-SexDifference.tsv", sep="\t", header=TRUE, row.names=1, quote="")
count <- as.matrix(count)
group <- factor(c("AM", "AM", "AM", "AF", "AF", "AF"), levels=c("AM", "AF"))
d <- DGEList(counts=count, group=group)
d <- calcNormFactors(d)
d <- estimateCommonDisp(d)
d <- estimateTagwiseDisp(d)
exact <- exactTest(d)
table <- as.data.frame(topTags(exact, n=nrow(data), sort.by = "none"))
write.table(table, file="FC-SexDifference.tsv", sep="\t", row.names = TRUE, col.names = TRUE, quote=F)

#GO ANALYSIS
table <- read.table("DEGandFC-SexDifference.tsv", sep="\t", header=T, row.names=1, quote="")
all.genes <- rownames(table)
is.degs <- all.genes[table$qvalue < 0.05 & abs(table$logFC) >= 0.5849625]

all.genes.entrez <- bitr(all.genes, fromType = "ENSEMBL", toType = "ENTREZID", OrgDb = "org.Mm.eg.db")
is.degs.entrez <- bitr(is.degs, fromType = "ENSEMBL", toType = "ENTREZID", OrgDb = "org.Mm.eg.db")

ego <- enrichGO(gene = is.degs.entrez[,2], universe = all.genes.entrez[,2], OrgDb = org.Mm.eg.db, ont = "ALL",
                pAdjustMethod = "BH", pvalueCutoff = 0.05, qvalueCutoff = 1, readable = TRUE)
ego.result <- simplify(ego)
write.table(ego.result, "GO-SexDifference.tsv", sep="\t", row.names=F, col.names = T, quote = F)

#KEGG pathway analysis
kegg.result <- enrichKEGG(gene=as.character(is.degs.entrez[,2]), universe=as.character(all.genes.entrez[,2]), keyType = "ncbi-geneid", organism="mmu",
         pvalueCutoff =  0.05, qvalueCutoff = 1, pAdjustMethod = "BH", use_internal_data = F)
write.table(kegg.result, "KEGG-SexDifference.tsv", sep="\t", row.names=F, col.names = T, quote = F)
```
